# A novel hybrid feature selection and ensemble-based machine learning approach for botnet detection

**DOI:** 10.1038/s41598-023-48230-1

**Published:** 2023-12-01

**Authors:** Md. Alamgir Hossain, Md. Saiful Islam

**Affiliations:** Institute of Information and Communication Technology (IICT), Bangladesh University of Engineering and Technology (BUET), Dhaka, 1000 Bangladesh

**Keywords:** Computer science, Information technology

## Abstract

In the age of sophisticated cyber threats, botnet detection remains a crucial yet complex security challenge. Existing detection systems are continually outmaneuvered by the relentless advancement of botnet strategies, necessitating a more dynamic and proactive approach. Our research introduces a ground-breaking solution to the persistent botnet problem through a strategic amalgamation of Hybrid Feature Selection methods—Categorical Analysis, Mutual Information, and Principal Component Analysis—and a robust ensemble of machine learning techniques. We uniquely combine these feature selection tools to refine the input space, enhancing the detection capabilities of the ensemble learners. Extra Trees, as the ensemble technique of choice, exhibits exemplary performance, culminating in a near-perfect 99.99% accuracy rate in botnet classification across varied datasets. Our model not only surpasses previous benchmarks but also demonstrates exceptional adaptability to new botnet phenomena, ensuring persistent accuracy in a landscape of evolving threats. Detailed comparative analyses manifest our model's superiority, consistently achieving over 99% True Positive Rates and an unprecedented False Positive Rate close to 0.00%, thereby setting a new precedent for reliability in botnet detection. This research signifies a transformative step in cybersecurity, offering unprecedented precision and resilience against botnet infiltrations, and providing an indispensable blueprint for the development of next-generation security frameworks.

## Introduction

A software program that executes automated operations through the Internet is known as a “bot”. Bots are meant to execute certain activities and may be built to automate operations such as web crawling, data scraping, and chatbot conversations. A network of hacked computers that is managed by a single attacker, known to as the bot master, is considered to as a botnet^1,2^. Each computer in the network, also known as a “bot”, is infected with malware that enables the botnet owner to control it remotely. The intruder can exploit the botnet to carry out a variety of harmful tasks, including sending phishing emails, abusing online services, initiating distributed denial of service (DDoS) attempts, damaging individuals, businesses, and governments by extracting private data, etc^3,4^.

Botnets are often created by infecting large numbers of computers through malware infections or phishing attacks. Once infected, the computers become part of the botnet and can be used to launch attacks on other computers or networks. Therefore, botnets pose a serious security threat to computer systems and networks by allowing malicious actors to gain unauthorized access and control of a large number of devices. From the report by Comparitech Limited, botnet attacks are increasing every seconds in the cyber space^5^. Detecting botnets is critical to ensure the security and integrity of computer systems and networks^6^. However, the ongoing evolution of botnets and their characteristics due to the advancement of technology has made it increasingly challenging to detect those using traditional methods^7^.

Machine learning-based approaches have shown promise in detecting botnets by analyzing network traffic patterns^8,9^. However, a single machine-learning algorithm may not be sufficient to detect all types of botnets effectively^10^. The quality of a single model can also decrease over time due to the ongoing evolution of botnets^11,12^. The utilization of multiple classifiers in some developed models for botnet detection has shown limitations, as they tend to yield comparatively lower detection rates and higher False Positive Rates (FPR)^13–15^. Imbalanced datasets pose a significant challenge to achieving accurate botnet detection. This imbalance in dataset distribution represents a major limitation in some existing research. Many existing datasets^16–19^ used in botnet detection contain correlated and mutually informed features, making feature selection a critical challenge. This underlines the pressing need for the development of innovative and relevant feature selection approaches, which can effectively identify and leverage the most informative features for enhanced botnet detection accuracy. This research suggests a potential strategy to enhance botnet identification by hybrid feature selection and ensemble-based machine learning approach. The aim is to increase the efficiency of detecting new and evolving botnets with higher TPR. Following is a list of the research’s main contributions:

- An in-depth evaluation of the strengths and limitations of existing botnet detection techniques.
- The development of an advanced feature selection technique to pinpoint relevant attributes for precise botnet identification.
- Developing an enhanced ensemble-based approach to significantly enhance the detection of botnets.
- Creation of a novel framework that combines chosen features with the developed ensemble-based machine-learning classifiers.
- Rigorous performance evaluation on publicly available benchmark datasets, showcasing the framework’s outstanding accuracy and low false positive rate.
- Implications for enhancing network security through continuous adaptation to emerging botnet threats.

The proposed approach has been empirically shown to achieve higher accuracy and better detection rates than existing single-method approaches. Our approach has the benefit of having the capability to quickly identify emerging botnets, which is essential for protecting networks, IoT ecosystems, and computer systems. The suggested technique for discovering and avoiding botnets involves multiple phases. To start, the most essential characteristics will be chosen using CA, MI, and PCA approaches. Next, utilizing ensemble methods like Extra-Trees, Bagging, Extreme Gradient Boosting, Stacking, and Random Forest, several learning algorithms will be integrated. The best ensemble based machine learning botnet detection methods will then be applied and various evaluation metrics will be calculated.

The subsequent phase of the research will encompass an in-depth literature review, offering a comprehensive summary of the pertinent studies conducted in the field. After that, each of the parts of the proposed framework will be explained. The experimental results, which provide convincing proof of the recommended strategy’s efficacy, will then be presented. We’ll do a thorough analysis of our proposed design, and we’ll use findings and data from practical tests to evaluate how well it performs. The primary results drawn from this research will be summed up in the conclusion section. We shall use a brief form with abbreviations throughout the paper to improve reading and clarity. The abbreviated terminology used in the article is included in Table 1 as a reference.

**Table 1:** Notations and abbreviations to enhance clarity and brevity.

Notations	Abbreviations
CA	Correlation analysis
RF	Random forest classifier
PCA	Principal component analysis
Adaboost	Ensemble Adaboost classifier
MI	Mutual information
DT	Decision trees
SVM	Support vector machines
PCA	Principal component analysis
KNN	K-nearest neighbors
NN	Neural networks
IRM	Interpolation reasoning method
DDoS	Distributed denial of service
WCC	World Competitive Contests Algorithm
DoS	Denial of service
DBO	Dung beetle optimizer
NN	Neural network
FRI	Fuzzy rule interpolation
TPR	True positive rate
AUC	Area under the ROC curve
FPR	False positive rate

## Literature review

Due to the rising threat that botnets represent to computer systems, networks and IoT devices, botnet detection has increased recently. For identifying botnets, a number of strategies have been suggested, including signature-based approaches, anomaly-based approaches, behavior-based approaches, and machine learning-based approaches. In this part, we examine the research on several botnet detection techniques.

Some research finds the efficacy of signature-based methods, which rely on predefined patterns to flag known botnet traffic. While this approach has demonstrated proficiency in recognizing established threats, it fundamentally lacks the flexibility to adapt to the polymorphic nature of contemporary botnets. The inherent limitation lies in its inability to detect novel or evolving botnets, which can easily circumvent detection by deviating from recognized signatures^20,21^. Anomaly-based detection, where statistical analysis serves to distinguish atypical traffic patterns indicative of botnet activity. While conceptually promising, this method is prone to a high rate of false positives, casting a shadow on its practical deployment. Moreover, the mutable nature of botnet attributes introduces additional complexities, as the system's calibrated norm can rapidly become obsolete, eroding the accuracy over time^22^. In the sphere of botnet detection, one study presents network traffic analysis as a cornerstone for identifying DDoS attacks—a prevalent botnet exploitation. This research harnessed the Random Forest (RF) algorithm, attaining an accuracy of nearly 98%. It particularly zeroed in on Command and Control (C&C) session identification to pinpoint botnet-fueled DDoS attacks. While the behavior-based detection employed offers valuable insights into botnet dynamics, its reliance on historical traffic behavior patterns presents a significant limitation. This method’s efficacy dwindles when confronted with novel botnet variants that have yet to establish a detectable behavioral footprint, underscoring the imperative for a more proactive detection paradigm that can anticipate and adapt to the evolving botnet landscape, which is the impetus driving the development of our novel hybrid feature selection and ensemble-based approach^23,24^.

The burgeoning domain of botnet detection has witnessed a significant shift toward machine learning-based methodologies, marked by training algorithms to discern and differentiate between benign and malicious traffic through historical data analysis. Such methods leverage the learned patterns to detect new botnets, drawing on a gamut of machine-learning algorithms like Support Vector Machines (SVM), Decision Trees (DT), K-Nearest Neighbors (KNN), and Neural Networks (NN). Despite the promise these techniques hold, their efficacy often wanes when singularly deployed against the multifaceted and sophisticated nature of modern botnets, as no lone algorithm proves universally potent. This reality begets the core motivation for our research—crafting a composite machine learning strategy that integrates the strengths of multiple algorithms to establish a robust, ensemble-based model capable of superior performance in the dynamic arena of botnet detection^12,25–29^. Ibrahim et al.^30^ proposed a multilayer architecture for botnet detection, employing the KNN algorithm and achieving an accuracy of 92%. Despite this, their approach manifested a notable false negative rate, and the detection efficacy was considered suboptimal. Dong et al.^31^ pioneered an Extreme Learning Machine (ELM)-based BotDetector, designed for swift botnet characteristic learning without extensive data processing. Their method stands out for its minimal resource utilization and quick detection capabilities. However, limitations such as low specificity, computational complexity, and a degree of unpredictability temper its practical applications. The FPR of this research is 0.02, which is higher for a large dataset. A brand-new IRM-based botnet detection technique was introduced in 2022 by Almseidin et al.^15^ for the detection of IoT botnets. They used the method in an ambiguous environment and achieved accuracy of about 96%. They recommended creating a hybrid machine learning-based technique to detect botnets for all contexts in future work. Another model by Sanjeetha et al.^32^ has a 97% accuracy rate. Only 25% of the data was used for training and 25% for testing. In addition, the FPR is greater in this model.

In 2018, Feng et al.^33^ presented an updated early detection method for distributed cyberattacks using RF that was based on machine learning and achieved 90% accuracy. They chose 10 features using PCA for the feature selection. Because C&C communication occurs during the planning stage of spread assaults, they attempted to identify it. Nevertheless, the detection strategy’s true positive rate is lower. With a 98% detection accuracy, Bansal and Mahapatra introduced clustering-based Machine Learning Approaches for Botnet Detection. Comparatively, the False Positive Rate (FPR) is relatively high^34^. Sobhanzadeh and Moghaddam introduced an innovative method merging Support Vector Machine (SVM) with Weighted Conditional Clustering (WCC) for botnet detection within IoT settings. They demonstrated that leveraging historical data of source and destination hosts, along with pertinent statistical metrics, can effectively differentiate between normal and anomalous traffic, achieving an accuracy of 97%. Despite its merits, the model displayed a True Positive Rate (TPR) that was below the average seen in other models^35^.

Ensemble learning combines many learning methods or models to enhance performance overall and minimize the danger of overfitting. To increase the precision and dependability of botnet detection, ensemble approaches have been employed. For the purpose of detecting botnets, some common ensemble approaches include extra-trees, bagging, extreme gradient boosting, and stacking may be used. Although boosting requires iteratively training models on misclassified data samples to increase their accuracy, bagging entails training several models on various subsets of the data and aggregating their predictions^36–38^. Stacking involves combining the predictions of multiple models using a meta-classifier^39^. With the use of decision trees and a famous ensemble technique called random forest, intrusion detection accuracy may be increased^40^. While Random Forests are powerful classifiers, their decision-making process can be challenging to interpret. A research paper on botnet forensic investigation using machine learning by Bijalwan^41^ was published in 2020. Where the decision tree classifier and boosting ensemble approach were employed by the author. His accuracy rate was around 98%. In this research, the authors recommended combining an ensemble classifier with a machine learning approach to analyze large amounts of data from botnet attacks. A hybrid strategy to choose characteristics and categorize cyberattacks was developed in Reference^42^. In that research, the correlation-based feature selection technique and the k-means clustering algorithm were used to obtain an optimum feature subset. The probabilistic Naive Bayes (NB) classification method and the decision tree were employed for the classification. Its disadvantage is that, due to its high false-positive rates, it is not a simple structure. Afrifa et al.^43^ demonstrated a method for precise and successful botnet attack detection in connected devices using ensemble machine-learning techniques. They employed the stacking ensemble technique and a decision tree classifier to identify botnets in computer network traffic with 99% accuracy. Despite its great accuracy, the stacking ensemble frequently uses more complicated models and may need more meta-model optimization rounds. This lengthens the time needed for testing and training. Moreover, this training and testing time is comparatively higher than other ensemble techniques^44^. Srinivasan et al.^13^ enhanced cyber security efforts by deploying an ensemble classification-based machine learning technique for botnet detection, achieving a notable accuracy of 94%. However, this approach was marked by a relatively high False Positive Rate (FPR) and a lower-than-desired True Positive Rate (TPR). Additionally, the implementation of their stacking ensemble method was time-intensive during the training and testing phases, posing constraints on efficiency.

The suggested botnet detection approach represents a considerable improvement over the single-classifier-based models’ limitations. The model exhibits higher performance across numerous publically available datasets by integrating hybrid feature selection strategies with an additional trees ensemble classifier. The model can take advantage of the combined intelligence of numerous decision trees by using an additional ensemble classifier for trees. By using an ensemble approach, the model is better able to handle the complex and varied patterns found in botnet traffic, which boosts its detection capabilities. The suggested model exhibits robustness across diverse publically available datasets, in contrast to standard single classifier-based models that are limited to particular datasets. Its capacity for generalization and adaptability to new, undiscovered botnet kinds is highlighted by its ability to perform well on various datasets. The model outperforms single-classifier-based techniques and shows a significant improvement in accuracy. Additionally, it achieves a greater True Positive Rate (TPR), demonstrating its capacity to correctly identify instances of botnets, while retaining a lower False Positive Rate (FPR), minimizing the number of occasions where regular traffic is mistakenly classified as botnet activity. In fact, the proposed model's increased performance metrics make it a very effective and strong tool for combating botnet threats across a variety of domains, such as the Internet of Things, Android, Industry 4.0, computer systems, and network environments.

## Proposed model developing

**Figure 1:**
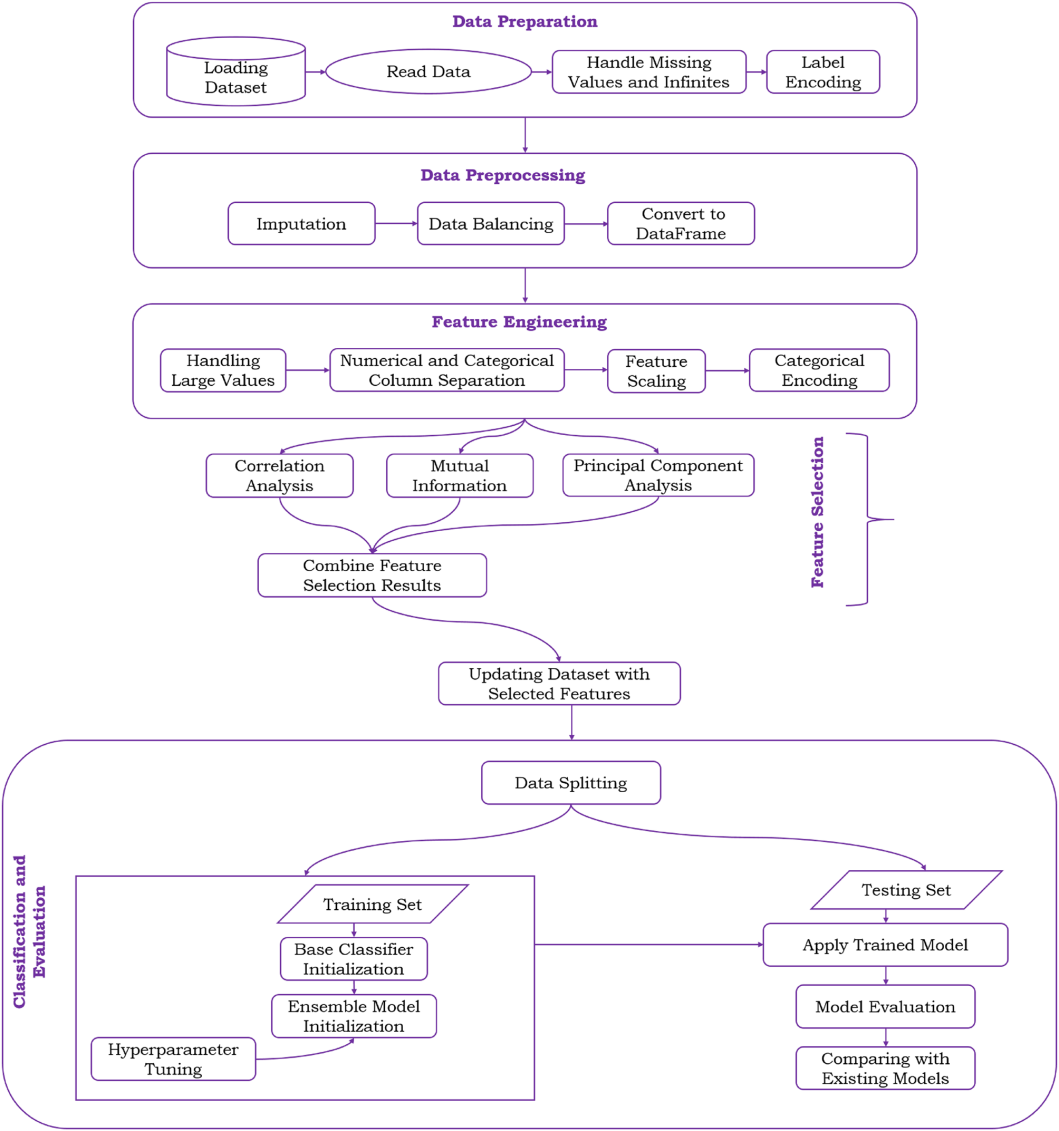
Proposed methodology for the detection of botnets.

The model development in machine learning enables intelligent systems to make accurate predictions, optimize processes, uncover hidden patterns, provide personalized experiences, handle large-scale data, and improve efficiency. Below is a detailed description of the proposed model. The development pipeline of the proposed model is given in Fig. 1.

### Dataset

The dataset plays a pivotal role in evaluating machine learning models and is a cornerstone of the entire model development process. A well-chosen dataset serves as the foundation for training, validating, and testing the model’s performance. It is instrumental in assessing the model’s capabilities, generalization ability, and potential to make accurate predictions on unseen data. In this research, an assortment of datasets has been employed to evaluate the proposed model thoroughly. Below is a short summary of the datasets that have been used.

#### N-BaIoT

A well-known and frequently used dataset in the area of botnet identification, particularly for Internet of Things (IoT) devices, is the N-BaIoT dataset^16^. It was carefully created from nine commercial Internet of Things (IoT) devices that had really been compromised by the well-known botnets Mirai and BASHLITE. The dataset’s emphasis on actual botnet infections in IoT devices makes it extremely pertinent for researching and comprehending the security issues unique to IoT ecosystems. IoT device vulnerabilities are frequently used by the Mirai and BASHLITE botnets to launch massive distributed denial-of-service (DDoS) assaults. With 116 features, the dataset delivers an enormous amount of information. These attributes, which are necessary for creating reliable botnet detection models likely include a wide range of characteristics, such as network traffic traits, communication patterns, and protocol-specific information. We took samples from the first IoT device out of the nine contaminated devices for our research. There were 1,018,298 samples in this subset. Table 2 lists the number of samples for each class. In the “Appendix” section, an explanation of each feature of the dataset is provided.

**Table 2:** Representation of classes after LabelEncoder application.

Numeric value	Class name	No. of samples
0	Benign	49,548
1	gafgyt_combo	59,718
2	gafgyt_junk	29,068
3	gafgyt_scan	29,849
4	gafgyt_tcp	92,141
5	mirai_ack	102,195
6	gafgyt_udp	105,874
7	mirai_scan	107,685
8	mirai_syn	122,573
9	mirai_udp	237,665
10	mirai_udpplain	81,982

#### Bot-IoT

The BoT-IoT dataset is a valuable tool created to support research on botnet detection and IoT (Internet of Things) security. It was extensively developed in the Cyber Range Lab at UNSW Canberra to produce a realistic network environment^17^. The dataset stands out due to its accurate depiction of an IoT network environment. In this environment, which closely resembles real-world IoT system environments, there is a mix of effective (normal) and malicious (botnet) relations. 46 features make up the dataset, and they offer crucial details regarding the peculiarities of network traffic. These features most likely consist of IoT-specific parameters, communication protocols, and other relevant information that help identify and categorize botnet activities. It is a comprehensive dataset that includes examples that are typical of many botnet kinds and covers a variety of botnet attacks. The information is carefully tagged to indicate the many types of botnet attacks that are there. Researchers and security professionals can create and assess models for IoT security, intrusion detection, and botnet mitigation using the BoT-IoT dataset. The dataset is a useful tool for comprehending and tackling security concerns in the developing Internet of Things space because it focuses on IoT devices and includes actual traffic scenarios.

#### CTU-13

Another well-known dataset for botnet identification and network security analysis is the CTU-13 dataset^18^. It originated in 2011 at CTU University with the main goal of supplying a sizable and varied collection of genuine botnet traffic mixed with regular background traffic. This dataset is crucial for testing and creating intrusion detection systems and botnet detection models. There are thirteen distinct captures or scenarios in the dataset. Each instance of botnet traffic represented by a scenario has particular characteristics, such as the type of malware utilized, the protocols employed, and the operations carried out. The dataset contains actual botnet traffic, which makes it useful for examining actual botnet behavior and comprehending the various strategies used by botnet operators. The CTU-13 dataset includes botnet traffic as well as regular and background traffic to replicate realistic network conditions. Researchers can create models that can differentiate between secure and harmful network activity due to this diversity. In our research, we employed about 1,525,249 samples, each of which was described by ten features. These properties of the network traffic, such as packet headers, flow statistics, or communication patterns, are mainly captured by these features.

#### ISCX

The ISCX dataset was designed to provide researchers access to a wide range of network traffic information for analyzing and discovering network intrusions, such as botnet activity. Data on network traffic was collected from a regulated network environment to construct the dataset. In addition to other sorts of attacks, such as but not limited to botnet attacks, it also contains benign (regular) traffic. It consists of 79 features that come from various facets of network traffic. These functions offer data on different network protocols, traffic patterns, and statistical characteristics. This dataset is frequently used, especially in the context of botnet detection, to assess the efficacy of intrusion detection models and approaches. The dataset is a great resource for researching botnet and network intrusion detection due to its comprehensiveness and labeled cases^19^.

#### CCC

The Cyber Clean Center (CCC) dataset is a commonly used publicly accessible dataset in the field of cybersecurity research. It has been used in many research studies to evaluate how well various models work at identifying and thwarting botnet-based assaults. The four separate sub-datasets in the dataset are C08, C09, C10, and C13. The CCC dataset includes traffic packets that are linked to particular port numbers within these sub-datasets. IRC (Internet Relay Chat) traffic is routed through port 6667, while HTTP (Hypertext Transfer Protocol) traffic is routed through port 80^45,46^.

This dataset, which consists of a total of 56 features, is a useful tool for developing and testing models intended to differentiate between botnet traffic and regular traffic. The dataset’s test feature divides traffic into two categories normal traffic and botnet traffic providing a foundation for evaluating the effectiveness of various detection and prevention methods.

#### CICIDS

The CICIDS dataset^47,48^ is a popular benchmark dataset used in the detection of botnets. It was created by the Canadian Institute for Cybersecurity and includes a significant amount of network traffic that was recorded in a genuine corporate network environment. The dataset contains both good and bad network traffic, including several botnet activities including DoS, port scanning, DDoS, and bot attacks. The dataset has been used to assess how well different deep learning and machine learning approaches can identify botnet traffic. Its use has resulted in the development of more accurate and efficient botnet detection systems that can help enhance the security of computer systems and networks.

### Experimental setup

In the experimental phase of this research, we established a computational environment using an 11th Gen Intel(R) Core(TM) i7-11,700 processor with 16 GB of RAM, running a 64-bit version of Windows 11 Pro. Our analyses were conducted using Python within the Jupyter Notebook interface, leveraging the powerful Scikit-learn library to implement machine learning models. We harnessed an array of Python libraries and methods to implement and evaluate our model. Key libraries included pandas for data handling, matplotlib for data visualization, imblearn.over_sampling for applying SMOTE to combat class imbalance, and sklearn.preprocessing for feature standardization and encoding with StandardScaler and LabelEncoder. Feature selection was conducted using sklearn.feature_selection’s SelectKBest with mutual_info_classif, as well as PCA from sklearn.decomposition for dimensionality reduction. The machine learning models were built using ensemble methods from Scikit-learn, such as ExtraTreesClassifier, XGBClassifier, RandomForestClassifier, BaggingClassifier, and StackingClassifier with decision tree and logistic regression base estimators. We also utilized SVC, DecisionTreeClassifier, GaussianNB, MLPClassifier, and XGBClassifier for a diverse set of predictive models. For model training and validation, the train_test_split method was integral in creating subsets of data. Specifically, we allocate only 20% of the data for testing purposes, reserving the remaining 80% for training. Model performance was quantified using a suite of evaluation metrics from sklearn.metrics, including accuracy_score, precision_score, recall_score, f1_score, confusion_matrix, cohen_kappa_score, roc_auc_score, and roc_curve. System performance and efficiency were monitored through the time and psutil libraries, ensuring an accurate log of model training and prediction times along with system resource utilization. These tools and methods collectively formed the backbone of our experimental framework, facilitating a thorough investigation into the efficacy of our novel botnet detection approach^49^.

### Data preprocessing

In this research, we utilized the mentioned Botnet Detection Datasets for our experiments. We started the preprocessing stage by checking for missing values and duplicates. Using the “duplicated()” function, we first examine the dataset for any duplicate entries. Duplicate rows are checked for and eliminated from the dataset. The “drop_duplicates()” function is used to achieve this, guaranteeing that each distinct data item is displayed only once. For many algorithms, infinity and extremely large values are difficult. Such values are substituted by NaNs (Not-a-Numbers) to address this problem. We substitute certain patterns, such as scientific notation or numeric strings, with NaN values using regular expressions. The dataset is then eliminated any rows with NaN values. To ensure data integrity and avoid mistakes during modeling, this step is essential. After that, label encoding is a process used to convert categorical labels into numerical values which is applied. Since most machine learning algorithms work with numeric data, categories must be mapped to numbers, making the data suitable for these models to process. The “LabelEncoder” class in scikit-learn performs this conversion by associating each unique category with a number.

The Synthetic Minority Over-sampling Technique, commonly abbreviated as SMOTE, represents a robust approach to mitigate the imbalance in datasets by generating synthetic data points. The principal strategy of SMOTE involves creating new examples that expand the minority class, effectively augmenting the dataset without the repetition involved in traditional over-sampling. This process is performed by creating synthetic minority class instances in the feature space neighborhood of existing minority examples, thereby enriching the dataset's diversity and aiding in the creation of more generalizable models. Let us denote our feature space as *X* with *n* features, where $X \in R^{m*n}$, and *m* is the number of instances. Correspondingly, the target variable *y* which denotes class labels is binary, with $y_{i} \in \left\{ {0, 1} \right\}$ here 0 represents the majority class and 1 represents the minority class. The goal is to balance the dataset by altering the distribution of *y* such that *y’* approaches a uniform distribution. For each minority class sample $x_{i}$, SMOTE calculates the *k-*nearest minority class neighbors. A synthetic instance is then generated by choosing one of the *k-*nearest neighbors, $x_{nn}$, and constructing a new instance $x_{new}$ as follows:

1 $$x_{new} = x_{i} + \lambda * \left( {x_{nn} - x_{i} } \right)$$

where $\lambda$ is a random number between 0 and 1. This operation constructs a new sample point that lies on the line segment between $x_{i}$ and $x_{nn}$ in the feature space. The procedure can be formally expressed through the following steps:

i. For a minority class instance $x_{i}$, identify its *k-*nearest neighbors using a chosen distance metric, typically Euclidean distance:

2 $$d\left( {x_{i} , x_{j} } \right) = \sqrt {\mathop \sum \limits_{l = 1}^{n} \left( {x_{il} - x_{jl} } \right)^{2} } \quad \forall_{j} \in \left\{ {1, \ldots ,m} \right\},\quad y_{j} = 1$$

ii. Randomly select *k′ neighbors from the k-*nearest neighbors, where $k^{\prime } \le k$.

iii. Generate synthetic instances using the interpolation formula given above for each selected neighbor.

iv. Repeat this process until the desired class proportion is achieved.

The dataset balancing through SMOTE in this research not only enhances the representation of the minority class but also contributes to a broader and potentially more accurate exploration of the feature space, resulting in a richer hypothesis space for the subsequent learning algorithms. This iterative refinement of the training set aims to yield a machine learning model with improved generalization capabilities across the botnet detection domain.

Overall, our preprocessing stage ensured that the dataset was clean and ready for feature engineering.

### Feature engineering

The feature engineering steps start with handling infinite values and large floats. All infinite values with NaN (Not a Number), making sure that no infinite or unreasonably large values that could skew the data are included. The regular expression targets strings formatted as floats (both in standard decimal and scientific notation) and integers, replacing them with NaN, which appears to be a mistake since it may inadvertently convert all numeric values to NaN. After replacing certain values with NaN, rows containing these NaNs are dropped from the dataset, ensuring the dataset has no missing or infinite values which could potentially cause errors during modeling. The “StandardScaler” normalizes the numerical columns by subtracting the mean and scaling to unit variance. This standardization of features is important since it ensures that each feature contributes equally to the distance computations in machine learning algorithms.

Normalization of numerical columns through the “StandardScaler” involves rescaling the distribution of values so that the mean of observed values is 0 and the standard deviation is 1. This process is known as Z-score normalization and can be mathematically represented as follows:

Let *X* be a matrix where each column represents a feature and each row represents an observation. The standard score (Z-score) for a single scalar value in a feature column can be calculated using the formula:

3 $$Z = \frac{x - \mu }{\sigma }$$

where *x* is the original value, *μ* is the mean of the feature column, *σ* is the standard deviation of the feature column.

For a $X_{j}$ feature column with n observations, the mean $\mu_{j}$ is calculated as:

4 $$\mu_{j} = \frac{i}{n}\mathop \sum \limits_{i = 1}^{n} x_{ij}$$

The standard deviation $\sigma_{j}$ for the same feature column is given by:

5 $$\sigma_{j} = \sqrt {\frac{i}{n - 1}\mathop \sum \limits_{i = 1}^{n} \left( {x_{ij} - \mu_{j} } \right)^{2} }$$

The “fit_transform” method of “StandardScaler” first computes $\mu_{j}$ and $\sigma_{j}$ for each feature in the fit phase. It then applies the above Z-score normalization to each element of the feature column in the transform phase, producing a new matrix where all features are standardized.

The processed dataset is now ready for the selection of relevant features.

### Feature selection and combining relevant features

In order to distinguish between botnet traffic and regular traffic, feature selection techniques including CA, MI, and PCA can assist discover the most organization capability in the dataset. CA^50^ measures the linear relationship between two variables. In botnet detection, it can identify features that have a high correlation with the target variable, which can help differentiate between botnet and normal traffic. MI^51^ is a statistical measure that quantifies the amount of information that one feature provides about another. Even if the connection is nonlinear, it may be used to find characteristics in botnet detection that have a substantial correlation with the target variable.

By transferring the data to a lower-dimensional space, the PCA approach decreases the dimensionality of the data. By locating the directions in the data that have the most variation, it can assist in identifying the most crucial properties. It can assist in botnet identification by locating the dataset’s most important properties, which can help distinguish between botnet traffic and regular traffic^52^. Algorithm 1 illustrates the algorithm for the selection of relevant features for the detection of botnets.

**Figure:**
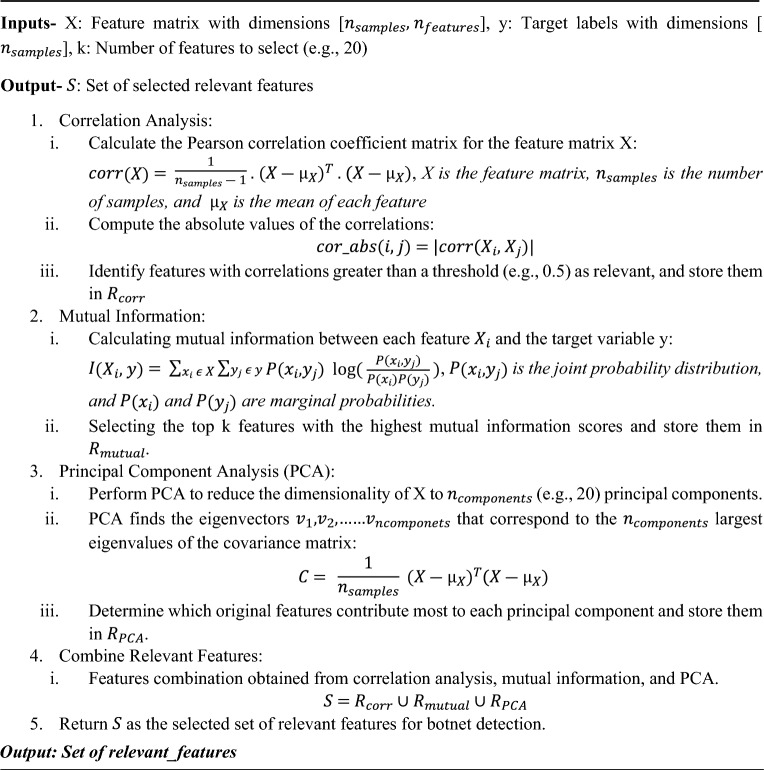
Algorithm 1 Selection process of relevant features

The three different feature selection techniques selects the most relevant features for botnet detection, which allowed us to achieve better performance in subsequent stages of our research.

### Ensemble method selection

Among the many ensemble approaches extra trees, extreme gradient boosting, bagging, random forest, and stacking are considered to evaluate the evaluation metrics in this research. Here is a brief overview of these ensemble methods:

#### Model with improved extra trees ensemble method

The Extra Trees ensemble classifier is a powerful machine learning technique for classifying traffic into multiple types of botnets or normal categories. It leverages the strength of multiple decision trees to achieve accurate and robust predictions. In the Extra Trees ensemble classifier, each decision tree is trained on a randomly selected subset of relevant features from the input dataset. The split points at each node are chosen based on maximizing the information gain or reducing the impurity criterion, such as Gini impurity or entropy^53,54^.

The model with the Extra Trees ensemble classifier effectively categorizes traffic instances into multiple types of botnets or normal classes. It can handle the complexity and diversity of different botnet types and generalizes well to new, unseen data, making it a versatile and reliable tool for traffic classification tasks. The process for the detection of botnets using extra trees ensemble is given in Algorithm 2.

**Figure:**
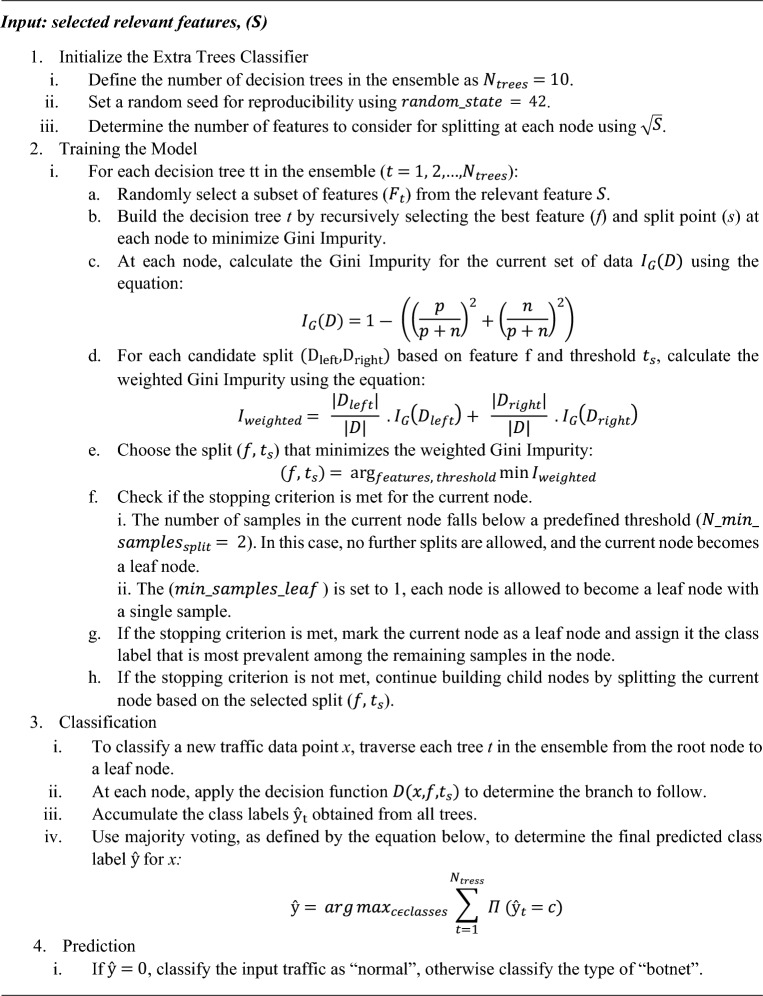
Algorithm 2 Process to classify traffics as botnet/normal using extra trees ensemble method

In the above algorithm, $I_{G} \left( S \right)$ is the Gini impurity for a set S, with p positive instances and n negative, $D\left( {x,f,t_{s} } \right)$ is a function with data point, feature, and threshold. ${\text{D}}\left( {{\text{x}},{\text{ feature}},{\text{ threshold}}} \right){ }$ returns “left” if ${\text{x}}\left[ {{\text{feature}}} \right] \le {\text{threshold}}$ and “right” otherwise. $I_{G, left}$ is the gini impurity for the left child node and ${\text{I}}_{{{\text{G}},{\text{right}}}}$ is the right child node based on feature tests.

The reason we chose to present the detection process in Algorithm 2 is due to the superior performance of the model with the Extra Trees ensemble. However, for the other ensemble-based approaches, we have provided the hyperparameters within the respective classifier descriptions.

#### Bagging ensemble method

The Bagging ensemble classifier is a powerful machine learning technique employed for classification tasks, designed to enhance predictive performance and reduce overfitting. It achieves this by creating an ensemble of multiple base classifiers, each trained on different bootstrap samples randomly drawn from the training dataset. The final prediction is determined through majority voting, where each base classifier's output contributes to the ultimate decision^37,55^.

For a classification problem like botnet detection, the final prediction ŷ in Bagging ensemble is obtained through majority voting:

6 $$\hat{y} = mode\left( {h_{t} \left( X \right)} \right)$$

where *ŷ* represents the final predicted class label (classification output) and *h*_*t*_*(X)* denotes the prediction of the *t*-th base classifier for the input features *X*. Each base classifier *h*_*t*_ produces a class label prediction based on the input features.

The number of base classifiers in the ensemble is used by n_estimators = 10. The size of bootstrap samples used for training by max_samples = 1.0, indicating that each classifier is trained on bootstrap samples of the same size as the original training set. The number of features considered during training for each base classifier with max_features = 1.0, all features are utilized during training. A boolean value determines whether bootstrap sampling is enabled (True) or not (False) with bootstrap = True, allowing bootstrap sampling. A boolean value indicates whether feature bagging is applied (True) or not (False). We used, bootstrap_features = False, to imply that feature bagging is not utilized. A random seed is used for reproducibility whose value is set to 42.

By harnessing the diversity of the base classifiers and reducing variance, Bagging provides an ensemble model that excels in capturing complex patterns within the data and yields more robust predictions compared to individual classifiers. The scikit-learn implementation, with its adjustable hyperparameters, offers flexibility in controlling the ensemble’s size, feature selection, and randomization process, empowering users to optimize the classifier for various classification tasks^56^.

#### Boosting ensemble method

XGBoost stands as a paragon of ensemble learning, renowned for its efficiency and prowess in handling diverse datasets with a sophisticated boosting mechanism^57^. It epitomizes an advanced gradient-boosting framework, employing a constellation of decision trees to progressively minimize errors and improve predictive accuracy, making it an invaluable tool for tackling intricate classification challenges such as botnet detection.

The XGBoost classifier’s training process for botnet detection utilizes an ensemble of decision trees constructed iteratively to correct the predecessors’ errors. The objective *L* at each iteration combines the loss function used to measure the prediction’s discrepancy from the true value and a regularization term to penalize model complexity, formally described as:

7 $$L\left( \theta \right) = \mathop \sum \limits_{i = 1}^{n} l\left( {y_{i} ,\hat{y}_{i} } \right) + \mathop \sum \limits_{k} \Omega \left( {f_{k} } \right)$$

where $y_{i}$ represents the true label for instance *i*, $\hat{y}_{i}$ is the corresponding prediction, and $f_{k}$ represents each decision tree in the ensemble. The regularization term $\Omega \left( {f_{k} } \right)$ is defined as:

8 $$\Omega \left( {f_{k} } \right) = \gamma T + \frac{1}{2}\lambda \left\| \omega \right\|^{2}$$

with *T* denoting the number of leaves in tree $f_{k}$, $\omega$ the scores on leaves, $\gamma$ he complexity cost added for each additional leaf, and $\lambda$ the L2 regularization term on the leaf weights. The hyperparameters utilized in the given code segment specify a learning rate (or “eta”) of 0.1, a fixed number of 10 estimators for the number of boosting rounds, and a max depth of 3 for individual trees. These hyperparameters dictate the learning trajectory.

#### Random forest ensemble method

To enhance the model’s overall performance, ensemble approaches mix numerous models. The ensemble approach is employed in this instance to detect botnets using the Random Forest algorithm. The number of estimators, the criterion, the minimum samples split, the maximum depth, the minimum samples leaf, and the maximum features are the initial hyperparameters for the Random Forest classifier. With this ensemble approach, the values of the evaluation metrics are likewise checked using the same four classifiers^40^.

For a given feature set *X* and labels *y*, the Random Forest classifier aims to construct *n* estimators, each a decision tree denoted by $f\left( {x, \Theta_{k} } \right)$, where, $\Theta_{k}$ are the random vectors independently sampled with the same distribution for all trees and *k* indexes through the ensemble of trees*.* The predictive function *F(x)* for a new sample *x* is obtained by averaging the predictions from all individual trees:

9 $$F\left( x \right) = \frac{1}{n}\mathop \sum \limits_{k = 1}^{n} f\left( {x,\;\Theta_{k} } \right)$$

The criterion “gini” refers to the Gini impurity, a measure of the frequency at which any element of the dataset will be mislabeled when it is randomly labeled according to the distribution of labels in the subset. Random Forest minimizes the total Gini impurity of individual trees.

In addition, our implementation initializes a Random Forest classifier called *“rf”* with 10 decision trees in this research. The default settings for the remaining hyperparameters have been chosen. When *“max depth”* is set to *“None”*, trees grow until all leaves are pure or have less samples than *“min samples split”* (whichever comes first). When *“min samples split”* is set to 2, a node is only split if it has at least two samples. Since *“min samples leaf”* is set to 1, a leaf node must include at least one sample. All samples are equally weighted since *“min weight fraction leaf”* is set at 0.0. When *“max features”* is set to *“auto”* the number of features that may be utilized at each split is limited to a value that equals the square root of the total number of features. When *“bootstrap”* is set to True, each decision tree is constructed using bootstrap samples. The out-of-bag score is not computed since *“oob score”* is set to False*. “n_jobs”* is set to *“None”,* which means that all CPUs are used. *“random_state”* is set to 42, which means that the same random state is used every time the classifier is trained. *“verbose”* is set to 0, which means that no messages are displayed during the training process. *“warm_start”* is set to False, which means that the previously trained trees are not used to initialize the new training. *“class_weight”* is set to *“None”*, which means that all classes are weighted equally. *“ccp_alpha”* is set to 0.0, which means that no pruning is performed. Finally, *“max_samples”* is set to *“None”*, which means that all samples are used to train each decision tree.

#### Stacking ensemble method

Training a meta-classifier to aggregate the predictions of various base classifiers is a step in the ensemble learning process known as stacking^58^. The meta-classifier chosen is Logistic Regression for this research, which is a linear classifier used to estimate probabilities of binary outcomes^39^. The Stacking classifier is created using a list of individual classifiers which are the same as the previous ensemble methods. Each individual classifier is trained on the same data and is expected to provide a different prediction. The Stacking ensemble classifier then combines these predictions from each individual classifier and utilizes the meta-classifier to arrive at a final prediction. The solver parameter specifies the algorithm used to optimize the loss function. In the implementation of this method LogisticRegression(solver = ‘lbfgs’, random_state = 42) is used, *“random_state* = *42”* sets the random seed to 42, so the results of the logistic regression model will be reproducible if the code is run again with the same value of 42 for *“random_state”.* This is essential for testing, troubleshooting, and contrasting various models. The *“lbfgs”* solver, which stands for Limited-memory Broyden-Fletcher-Goldfarb-Shanno algorithm, is employed in this situation. It is a quasi-Newton approach that seeks for the minimum of the loss function by approximating the Hessian matrix, which is the second derivative of the loss function^59^.

### Assessment metrics employed to evaluate the model’s effectiveness

Utilizing a variety of assessment criteria, we have demonstrated the efficacy of our suggested approach in detecting botnets. These metrics are succinctly outlined as follows:

#### Accuracy

A frequent assessment statistic in machine learning to assess the effectiveness of a classification model is accuracy. It evaluates the percentage of correctly categorized cases among all instances^60^.

The accuracy is computed by dividing the total number of occurrences (TP + TN + FP + FN) by the number of cases that were properly classified

10 $$Accuracy = \frac{{\left( {TP + TN} \right) }}{{ \left( {TP + TN + FP + FN} \right)}}$$

here True Positives (TP) (instances that are actually positive and are correctly predicted as positive), True Negatives (TN) (instances that are actually negative and are correctly predicted as negative), False Positives (FP) (instances that are actually negative but are predicted as positive), False negatives (FN) Situations where a positive outcome is predicted but truly happens).

#### Precision

Another frequently used evaluation parameter in machine learning to evaluate the efficacy of a classification model is precision. Of all occurrences that were expected to be positive, it calculates the percentage of genuine positives. As it implies a larger proportion of real positives among all instances predicted as positive, a higher accuracy score is preferable. Precision levels vary from 0 to 1, with 0 indicating no precision and 1 representing complete precision (all cases predicted as positive are really positive) (all instances predicted as positive are actually negative)^60^.

The accuracy is computed by dividing the total number of instances predicted as positive (TP + FP) by the number of true positives (TP):

11 $$Precision = \frac{TP }{{ \left( {TP + FP} \right)}}.$$

#### Recall

Another assessment metric, recall estimates the percentage of real positives among all positive examples^60^. Also known as the True Positive Rate (TPR).

The recall is derived by dividing the total number of real positive instances (TP + FN) by the number of true positives (TP):

12 $$Recall = \frac{TP }{{ \left( {TP + FN} \right)}}.$$

A higher recall value is better, as it indicates that the model is correctly identifying more actual positive instances.

#### F1-score

Machine learning uses the F1-score as an assessment parameter to evaluate the trade-off between recall and accuracy. Its range is 0 to 1, with 1 indicating the ideal balance between precision and memory. It is the harmonic mean of precision and recall^61^.

Calculating the F1-score involves dividing the total of the accuracy and recall outputs by their product:

13 $$F1{ - }score = 2*\frac{{\left( {precision * recall} \right) }}{{\left( {precision + recall} \right)}}.$$

#### Cohen’s Kappa

Cohen’s Kappa is frequently used to assess how well a classification model is working. The observed agreement between the model’s predictions and the actual labels is compared to the predicted agreement that would happen by chance to determine Cohen’s Kappa. A model is considered to be effective if the observed accuracy value exceeds the expected accuracy^62^. The formula for Cohen’s Kappa is as follows:

14 $$Kappa = \frac{{\left( {Po - Pe} \right)}}{{ \left( {1 - Pe} \right)}}$$

here *Po: the percentage of actual labels that agree with predictions from the model, Pe: the percentage of anticipated concordance between the model’s predictions and the actual labels as determined by chance*.

#### The values of Po and Pe can be calculated as follows:

$$Po = \left( {true\;positives + true\;negatives} \right)/\left( {true\;positives + true\;negatives + false\;positives + false\;negatives} \right)$$

$$\begin{aligned} Pe & = ((true\;positives + false\;positives)*(true\;positives + false\;negatives) \\ & \quad + \;(false\;positives + true\;negatives)*(false\;negatives + true\;negatives))/(true\;positives + true\;negatives + false\;positives + false\;negatives) ^ {2} . \\ \end{aligned}$$

#### Area under the ROC curve

Machine learning models frequently use the performance metric AUC^63^. The TPR vs FPR for various categorization criteria is plotted on the ROC (Receiver Operating Characteristic) curve.

The area under the ROC curve, or AUC, is a binary classification problem's measure of how separable the positive and negative classes are. The AUC goes from 0 to 1, where 0 denotes a model that is flawlessly awful and consistently predicts the incorrect class and 1 denotes a model that is flawlessly good and consistently predicts the right class. A random model with an AUC of 0.5 is no better than guessing^64^. The AUC score is calculated by the Eq. (9).

15 $$AUC \, = \frac{{s_{p} - n_{P} \left( {n_{n} + 1} \right)/2}}{{n_{p} \cdot n_{n} }}$$

where s_p_ is the sum of all the ranked positive instances, and n_p_ and n_n_ stand for the number of positive and negative examples, respectively.

A high AUC shows that the model is good at differentiating between positive and negative classes. Because it is indifferent to both the selected classification threshold and the class imbalance in the data, it is a valuable statistic. AUC may also be used to compare how several categorization models perform on the same dataset.

#### Balanced accuracy (BACC)

The BACC metric is an important indicator for assessing a model's classification performance, especially when the dataset shows class imbalances. In order to provide an overall evaluation of a model’s correctness, it balances its sensitivity (True Positive Rate) and specificity (True Negative Rate) across all classes. The following equation is used for calculating the BACC:

16 $$BACC \, = \frac{1 }{{ 2 }}\left( {\frac{TP }{{ \left( {TP + FN} \right)}} + \frac{TN }{{ \left( {TN + FP } \right)}}} \right).$$

The other metric like the Error Rate is a metric that quantifies the proportion of misclassified instances in a classification model. Lower Error Rate indicates better model performance. Training Accuracy measures how well a model performs on the data it was trained on. High Training Accuracy suggests the model has learned from the training data. Testing Accuracy evaluates a model’s performance on a separate, unseen dataset (the test set). High Testing Accuracy indicates the model’s ability to generalize to new, unseen data. Through the Eqs. (11) to (13) provided below, error rate, training accuracy, and testing accuracy are all measured.

17 $$Error\;rate = 1{-}Accuracy$$

18 $$Training\;accuracy = Correctly\;predicted\;instances/total\;instances\;in\;training\;data$$

19 $$Testing\;accuracy = Correctly\;predicted\;instances/total\;instances\;in\;test\;data$$

## Result and discussion

In the following section, we evaluate the effectiveness of the approach we suggested for detecting botnets. In order to visualize the model, we first use the N-BaIoT dataset and the extra trees ensemble technique. The “Appendix” section contains a full description of each of the 116 features that make up the N-BaIoT dataset. We use 1,018,298 initial samples in total from this dataset for our experiment.

The label column in the N-BaIoT dataset encounters eleven different types of values, including benign, gafgyt_junk, mirai_udp, mirai_syn, mirai_scan, gafgyt_udp, mirai_ack, gafgyt_tcp, mirai_udpplain, and gafgyt_combo. Among these, “benign” indicates normal flows, whereas each of the other categories is botnets. Using a variety of evaluation criteria, comparisons to other datasets, use of various ensembles, we examine the model’s performance.

To record the outcomes of the model’s application across diverse datasets, we employ the identical procedures elaborated extensively in the section titled “Proposed Model Development”, By changing simply the datasets, the model’s effectiveness is being evaluated together with its potential for botnet detection. As a result, we evaluate the effectiveness of our model’s botnet detection by comparing it with other botnet detection models that are currently available.

### Analysis of the findings

**Figure 2:**
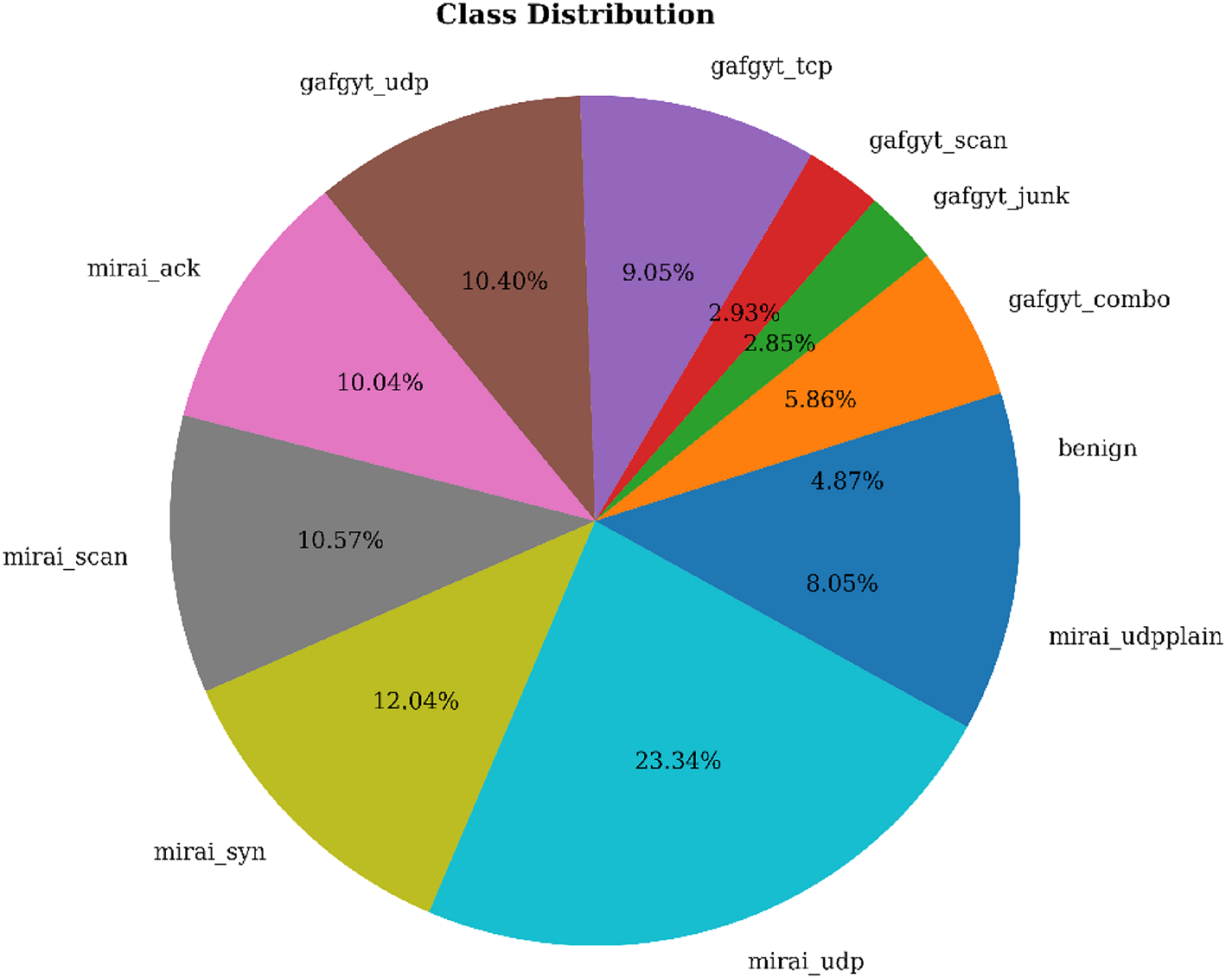
Class distribution of the initial dataset.

The dataset’s class distribution is illustrated in Fig. 2 and includes eleven different class types. The percentage of each class is shown within the relevant pie slice, which represents each class as a slice. The picture gives a fast grasp of the relative proportions of each class by visualizing how the dataset is distributed throughout the various classifications.

After category labels were converted into numerical representations using the LabelEncoder(), the classes are shown in Table 2. A distinct integer number between 0 and 10 is given to each class. The table provides a concise representation of the original class labels and their corresponding encoded values.

**Figure 3:**
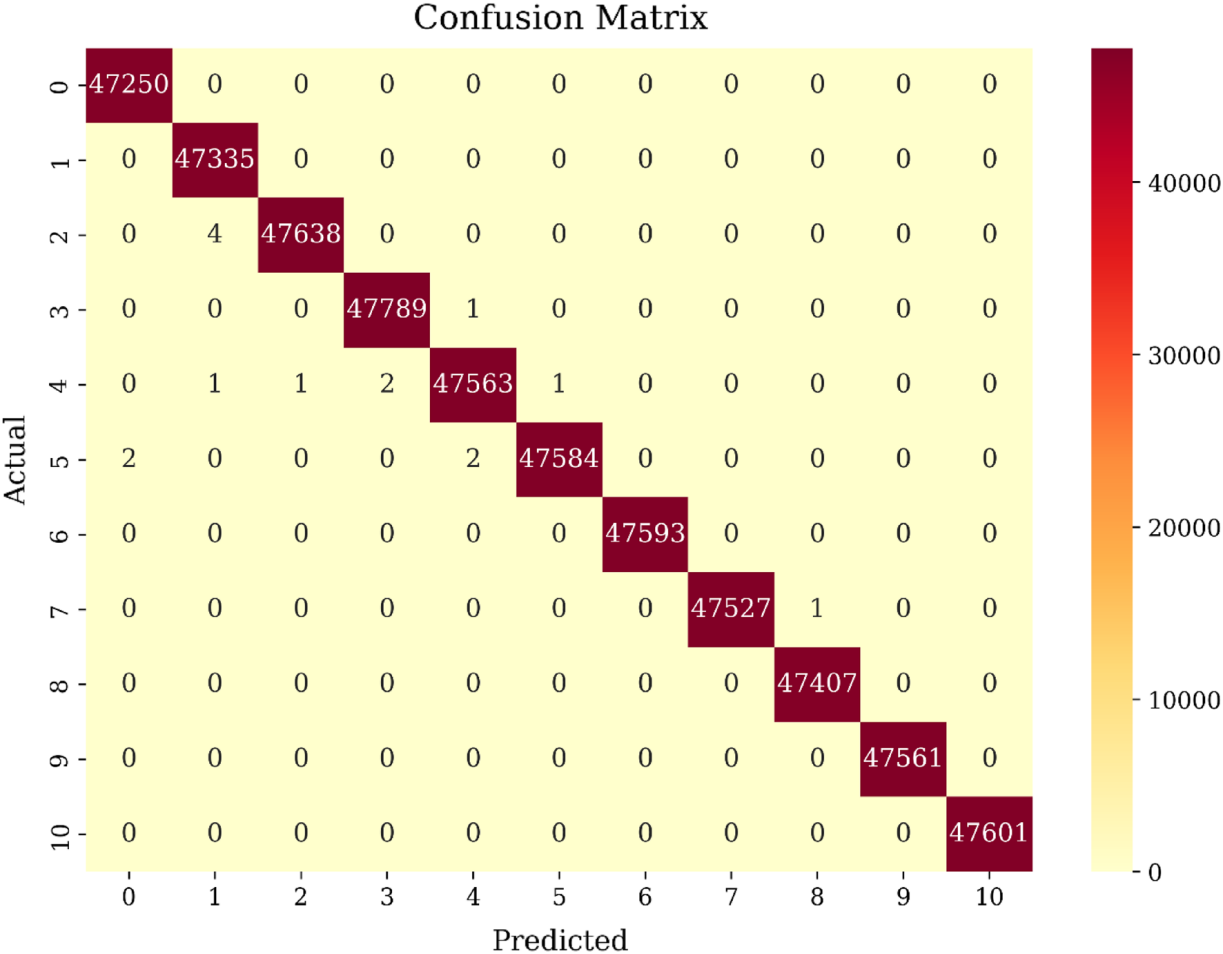
Visualization of confusion matrix.

Following the application of the SMOTE technique, each category within the target column is represented by 237,665 samples, achieving a balanced dataset. Figure 3 presents the confusion matrix derived from evaluating the model on test data (20%). Each cell in the heatmap represents the count of predictions made by the model for each class. The y-axis displays the actual labels, while the x-axis displays the predicted labels. Annotating each cell with the respective count provides a clear and comprehensive overview of the model’s performance and the distribution of predictions across different classes. Based on the computations in the figure and Table 3, the model performs effectively as shown by the large number of True Positives (TP) in various classes. The considerable counts of True Positives indicate that the model successfully identified and labeled instances belonging to their respective classes, such as gafgyt_combo, gafgyt_junk, gafgyt_scan, mirai_ack, and others.

**Table 3:** values for various classes in the confusion matrix.

Test data (20%)	TP	FP	FN	TN
Class 0	47,250	2	0	475,611
Class 1	47,335	5	0	475,523
Class 2	47,638	1	4	475,220
Class 3	47,789	2	1	475,071
Class 4	47,563	3	5	475,292
Class 5	47,584	1	4	475,274
Class 6	47,593	0	0	475,270
Class 7	47,527	0	1	475,335
Class 8	47,407	1	0	475,455
Class 9	47,561	0	0	475,302
Class 10	47,601	0	0	475,262

Examining the False Positives (FP), which represent instances incorrectly classified as positive, we observe relatively low counts across most classes. This suggests that the model has a reasonably low rate of misclassifying instances as positive when they actually belong to a different class. The False Negatives (FN), show minimal counts or even zero for most classes. This implies that the model has a strong ability to correctly identify instances that do not belong to a specific class. In addition, the True Negatives (TN), which represent instances correctly classified as negative, we observe high counts across the majority of classes. This indicates the model’s proficiency in accurately identifying instances.

According to the confusion matrix, the average True Positive Rate (TPR) of 99.99% indicates that the model has a nearly perfect ability to correctly identify positive instances across all classes. Moreover, the average False Positive Rate (FPR) of 0.00% suggests that the model has an extremely low rate of falsely classifying negative instances as positive.

The evaluation metrics for each class in our multiclass classification model for various forms of botnets are shown in Table 4. The model performs exceptionally well across all classes, showing excellent values for F1-score, recall, accuracy, and precision, all at 1.0000 (100%). This means that the model successfully categorizes each class with almost perfect accuracy, demonstrating a very trustworthy and powerful prediction capacity.

**Table 4:** Evaluation metrics for each class.

Class	Accuracy	Precision	Recall	F1-score
0	1.0000	1.0000	1.0000	1.0000
1	1.0000	0.9999	1.0000	0.9999
2	1.0000	1.0000	0.9999	0.9999
3	1.0000	1.0000	1.0000	1.0000
4	1.0000	0.9999	0.9999	0.9999
5	1.0000	1.0000	0.9999	0.9999
6	1.0000	1.0000	1.0000	1.0000
7	1.0000	1.0000	1.0000	1.0000
8	1.0000	1.0000	1.0000	1.0000
9	1.0000	1.0000	1.0000	1.0000
10	1.0000	1.0000	1.0000	1.0000

Due to its extraordinary effectiveness, it is a trustworthy and beneficial tool for multiclass classification-based botnet identification in a variety of real-world circumstances.

The evaluation metrics (weighted) for the various ensemble approaches for botnet detection employed in this research are shown in Table 5. The research compares Extra Trees, Random Forest, Bagging, Extreme Gradient Boosting, and Stacking as the five ensemble approaches. The model with the Extra Trees Ensemble approach stands out as the best performer for botnet identification among the ensemble methods examined. It has almost flawless scores in Accuracy, Precision, Recall, and F1-score, demonstrating an amazing capacity to correctly identify botnet occurrences while reducing false positives. Notably, the Extra Trees model has a remarkable FPR of 0.0000, making it the most accurate at differentiating between botnets and regular instances. These results demonstrate the Extra Trees Ensemble method’s superiority over the other methodologies under investigation and demonstrate its potential as a highly efficient and reliable solution for actual botnet detection scenarios.

**Table 5:** Evaluation metrics (average) − 1 for various ensemble techniques checked in this research.

Accuracy	Precision	Recall	F1-score	FPR
Model with extra-trees ensemble technique				
0.9999	0.9999	0.9999	0.9999	0.0000
Model with bagging ensemble technique				
0.9091	0.9507	0.9091	0.8788	0.0090
Model with random forest ensemble technique				
0.9091	0.9491	0.9091	0.8789	0.0091
Model with extreme gradient boosting ensemble technique				
0.9992	0.9992	0.9992	0.9992	0.0000
Model with stacking ensemble technique				
0.9090	0.9422	0.9090	0.8788	0.0091

The average error rate, BACC, training accuracy, and testing accuracy for several ensemble approaches used in this botnet detection model are shown in Table 6 as assessment metrics. The model using the Extra Trees Ensemble technique has the greatest Balanced Accuracy (BACC) score of 0.9999, demonstrating its ability to correctly identify instances as belonging to botnets or not even when the data is unbalanced. The model’s accuracy in creating accurate predictions is demonstrated by the incredibly low Error Rate of 0.0000, underscoring its dependability for botnet-detecting jobs. A Training Accuracy of 1.0000, indicating the Extra Trees approach’s capacity to precisely match the training data, is also achieved. Additionally, the high Testing Accuracy score of 0.9999 suggests that the model can generalize well to unseen data, making it a robust solution for real-world botnet detection scenarios.

**Table 6:** Evaluation metrics (average) − 2 for the model.

BACC	Error rate	Training accuracy	Testing accuracy
Model with extra trees ensemble method			
0.9999	0.0000	1.0000	0.9999
Model with bagging ensemble method			
0.9500	0.0909	0.9092	0.9091
Model with random forest ensemble method			
0.9500	0.0909	0.9092	0.9091
Model with extreme gradient boosting ensemble method			
0.9995	0.0007	0.9993	0.9992
Model with stacking ensemble method			
0.9500	0.0909	0.9091	0.9090

The average AUC Score, Cohen’s Kappa, Observed Accuracy (Po), and Expected Accuracy (Pe) for several ensemble approaches are shown in Table 7 as other assessment metrics. With a Cohen’s Kappa of around 0.9999, the model with the Extra Trees Ensemble approach shows outstanding agreement between its predictions and the actual classifications. Its outstanding capacity to precisely detect botnets is indicated by its high Observed Accuracy (Po) and AUC Score of 0.9999 and 1.0000, respectively. Additionally, the Expected Accuracy (Pe) is substantially lower at 0.0909, emphasizing that the model’s performance is significantly better than random chance. The findings highlight the model with the Extra Trees model’s potential as a powerful and reliable tool for identifying botnets, making it an excellent option for cybersecurity applications.

**Table 7:** Evaluation metrics (average) − 3 for the model.

Cohen’s Kappa	Observed accuracy (Po)	Expected accuracy (Pe)	AUC score
Model with extra trees ensemble method			
0.9999	0.9999	0.0909	1.0000
Model with bagging ensemble method			
0.8999	0.9090	0.0909	0.9909
Model with random forest ensemble method			
0.9000	0.9091	0.0909	0.9909
Model with extreme gradient boosting ensemble method			
0.9991	0.9992	0.0909	1.0000
Model with stacking ensemble method			
0.8999	0.9090	0.0909	0.9909

Overall, the proposed model with the Extra Trees Ensemble approach shows to be an excellent choice for botnet identification based on the assessment criteria shown in the Tables 5, 6 and 7. Because of its remarkable accuracy, precision, and generalization abilities, it has the potential to be an effective tool for enhancing cybersecurity and preventing the widespread issue of botnets.

**Figure 4:**
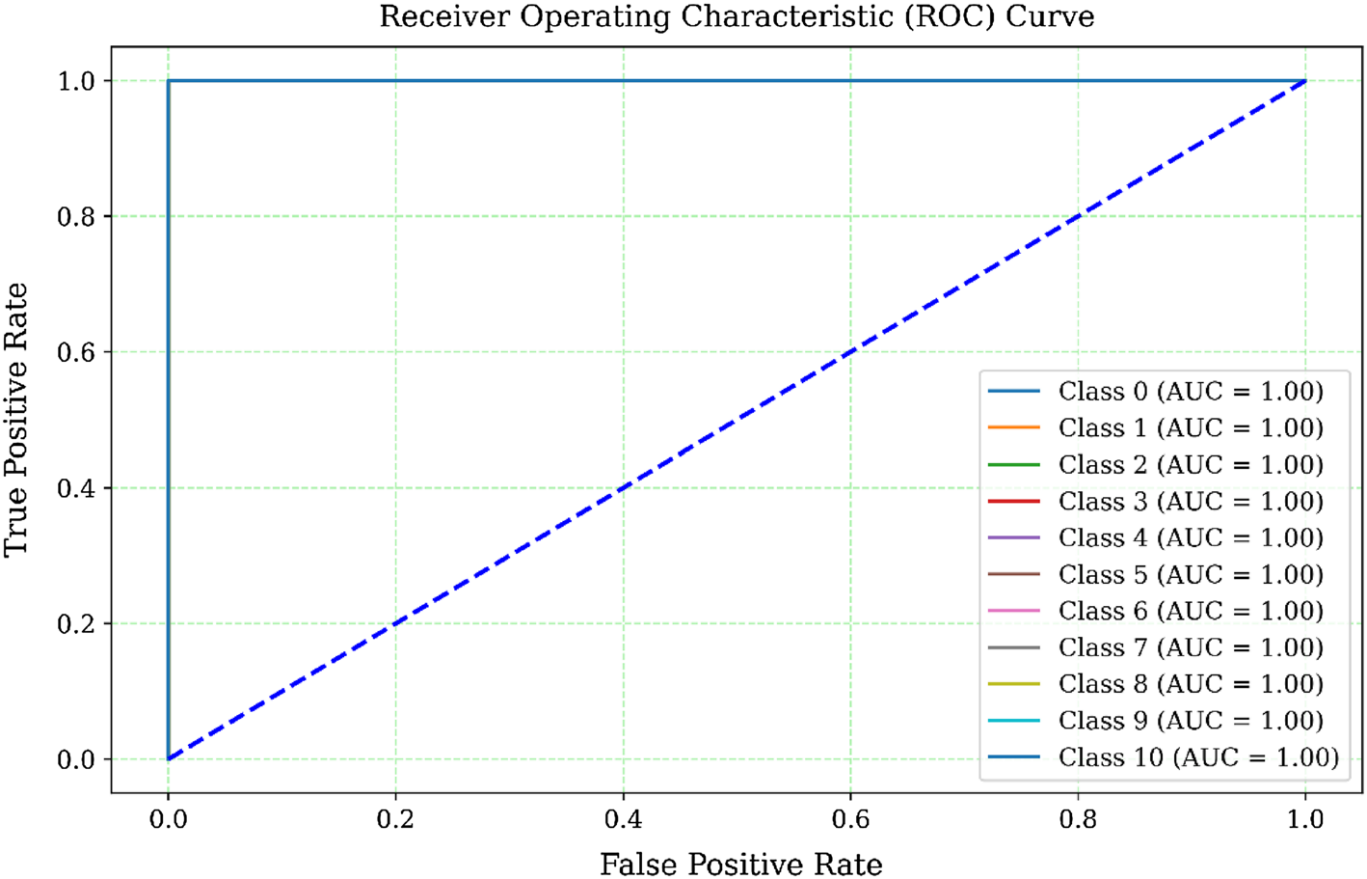
ROC Curve for different classes.

Figure 4 shows the ROC Curve of TPR against FPR for various classes. The TTPR and the FPR connection at various thresholds is depicted in this picture by a ROC curve. With an AUC score of 1, the model is considered to have almost flawless classification performance and very high discriminatory power. According to the ROC curve, the model is very capable of differentiating between positive and negative classes.

We may therefore draw the conclusion that the suggested model with extra trees is very accurate and can successfully and confidently detect botnet attacks.

**Figure 5:**
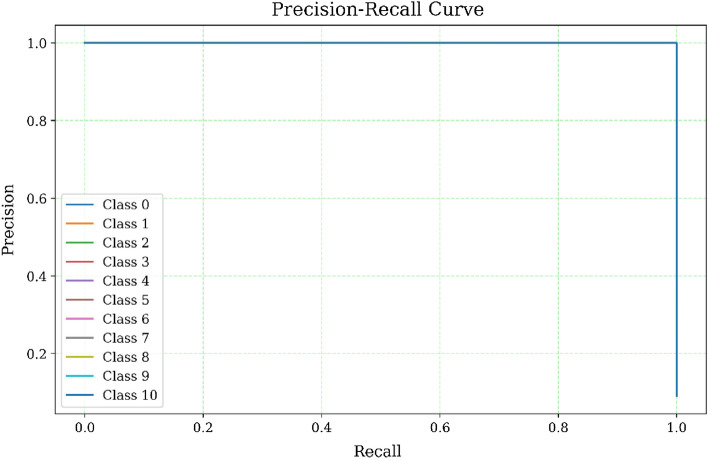
Visualization of precision recall curve.

A precision-recall curve, shown in Fig. 5, illustrates the trade-off between accuracy and recall for various threshold levels. This curve visually illustrates the alterations in precision and recall values when adjusting the decision threshold for classifying samples. On the x-axis, we observe the recall, which is synonymous with the true positive rate or the percentage of actual positive cases correctly identified as positive by the model. This graphical representation offers insights into the model’s capacity to differentiate between distinct classes, enabling a side-by-side comparison of precision and recall values for each class.

Since the precision and recall are both 99.99%, the curve should show a high precision and recall rate throughout, indicating that the model is performing very well. A perfect precision-recall curve would be a right angle, going up to the top at the perfect recall of 1, then continuing horizontally at the perfect precision of 1. If the curve is a straight line, it would mean that the model’s precision and recall rates are consistent throughout different threshold values. Therefore, based on the precision-recall curve, we can infer that the model is performing exceptionally well, achieving very high precision and recall rates for different threshold values.

**Figure 6:**
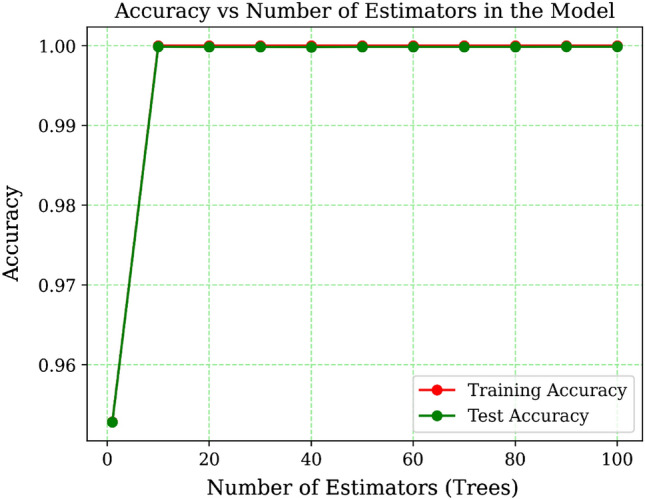
Accuracy of the model for different no. of trees.

Figure 6 presents a graphical representation illustrating the accuracy variations of an Extra Trees Classifier model across various ensemble sizes. On the x-axis, you can observe the number of trees in the ensemble, while the y-axis represents the model’s precision. The plot comprises two distinct lines: one representing test accuracy and the other representing training accuracy. The test accuracy line provides insights into the model’s performance on the test dataset, while the training accuracy line indicates how accurately the model fits the training data.

Based on the findings, it’s evident that as the number of trees in the model increases, both the training accuracy and test accuracy converge towards a value of 1.0, or very close to it. This suggests that the model is proficient at accurately classifying the data. The training accuracy begins at roughly 0.99 and swiftly reaches 1.0 when employing 10 trees as estimators, maintaining a similar level with further increases in estimators. This underscores the model’s exceptional fit to the training data, achieving near-flawless accuracy. Simultaneously, the test accuracy commences at a high level of approximately 0.99 and gradually nears 1.0 as the number of trees grows. This indicates that the model demonstrates robust generalization to new, unseen data, as the accuracy on the test set consistently improves with the addition of more trees.

Overall, the generated figure demonstrates that the Extra Tree Classifier model is highly effective for the detection of botnets. The accuracy values are consistently high, both on the training and test sets, indicating that the model is able to accurately classify instances of botnet behavior.

### Comparison of the proposed models’ evaluation metrics against the ones of recent models

The proposed model displays outstanding performance on multiple datasets comparing existing models, as depicted in Table 8. The table only contains published models with datasets covering the years 2020–2023. On the N-BaIoT dataset, the model achieves near-perfect accuracy, recall, precision, and F1-score, all reaching 99.99%. This exceptional accuracy demonstrates the model’s ability to accurately classify both positive and negative instances, making it highly reliable for detecting botnets in this dataset. Similarly, on the Bot-IoT dataset, the proposed model achieves perfect scores of 100.00% in all evaluation metrics, indicating its remarkable precision and recall in distinguishing botnet activities from normal traffic. The proposed model consistently delivers remarkable results not only on the N-BaIoT and Bot-IoT datasets but also on other datasets, including CTU-13, ISCX, CICIDS, and CCC. Its exceptional performance across diverse datasets underscores its effectiveness in detecting botnet activities and reinforces its position as a powerful and reliable solution for enhancing cybersecurity measures. Comparing the proposed model with other existing models on the same datasets reveals its superiority in botnet detection.

**Table 8:** Comparative performances of different models with different datasets.

Used dataset	Model and year with references	Accuracy (%)	Precision (%)	Recall (%)	F1-score (%)
N-BaIoT^16^	Proposed model	99.99	99.99	99.99	99.99
	LDL, 2023^65^	98.37	83.31	86.32	84.47
	FGOA-kNN, 2023^66^	98.07	97.04	98.73	97.87
	SGDC, 2023^67^	*	98.43	98.42	98.41
	ER-VEC, 2023^14^	95.64	*	*	*
	WCC and SVM, 2022^35^	96.70	94.90	94.70	94.80
	CNN-LSTM, 2022^68^	*	94.00	89.00	85.00
	BGWO, 2022^69^	98.97	*	*	*
	LGBA-NN, 2021^70^	90.00	85.23	90.00	86.64
	RNN, 2021^71^	89.75	*	*	*
	CART, 2020^72^	99.00	*	*	*
Bot-IoT^17^	Proposed model	100.00	100.00	100.00	100.00
	CTGAN with MLP, 2023^73^	98.93	99.84	98.93	99.07
	SOPA-GA-CNN, 2023^74^	98.20	97.67	97.75	97.71
	Modified SVM, 2023^75^	97.00	97.00	97.00	97.00
	DBO-Catboost, 2023^76^	96.10	96.20	96.10	96.10
	BTC-SIGBDS, 2023^77^	94.98	*	*	*
	SGDC, 2023^67^	*	92.28	92.48	92.37
	Fuzzy interpolation, 2022^15^	96.41	98.80	98.80	98.80
	FRI, 2021^78^	95.40	96.00	96.00	96.00
	C4.5, 2020^79^	97.62	97.63	99.99	98.79
CTU-13^18^	Proposed model	98.77	98.66	98.77	98.71
	DT, 2023^80^	92.21	92.21	92.21	92.21
	IGWO, 2022^81^	98.87	99.15	93.45	*
	MLC, 2021^82^	97.00	98.10	99.60	98.00
	KNN with SMOTE, 2021^30^	92.05	97.90	84.80	90.88
	RNN, 2021^83^	*	87.10	97.00	91.80
	LR, 2021^84^	*	66.80	92.40	77.54
	KNN, 2020^85^	94.20	*	*	*
	Multi-layer with DT^26^	98.70	*	*	*
	DT, 2019^25^	94.40	*	*	*
ISCX^19^	Proposed model	99.95	99.95	99.95	99.95
	Byte histograms and HOG descriptors with Extra Trees, 2022^86^	97.50	98.00	98.00	98.00
	AE NN with DT 2022^87^	99.20	99.10	99.10	99.10
	ELM, 2021^31^	98.67	*	99.00	*
	AdaBoost-DT, 2020^41^	98.36	98.85	98.23	98.54
CCC^45^	Proposed model	99.75	99.51	100.00	99.75
	PSO and GA, 2023^88^	97.30	*	91.60	86.00
	Stacking ensemble, 2023^13^	94.08	71.42	86.50	78.24
	KNN, 2021^82^	97.00	98.10	99.60	98.00
CICIDS^47^	Proposed model	99.99	99.99	99.99	99.99
	RF, 2023^89^	99.00	96.00	91.00	93.00
	NN, 2022^90^	98.58	96.67	97.15	96.21
	Bi-GRU NN, 2022^91^	99.30	99.00	99.30	99.60
	NBMT, 2022^92^	98.94	*	*	*
	RF, 2020^93^	99.96	99.20	97.00	98.10

(*) means not mentioned.

The proposed scheme for botnet detection surpasses other existing approaches for all evaluation metrics and displays impressive performance across a variety of datasets. Its outstanding results validate its effectiveness in detecting botnet activities and highlight its potential as a robust solution for enhancing cybersecurity measures against botnet threats.

### The proposed models accuracy with computational complexity for various datasets

Table 9 presents the results of the model on different botnet datasets. The table provides accuracy scores and other performance metrics including TPR, Training Accuracy, Testing Accuracy, and AUC Score. The training accuracy is consistently high, being 100.0% for all datasets, suggesting that the model has learned the training data very well and does not suffer from overfitting. The testing accuracy is also very high, indicating that the model generalizes effectively to unseen data. The TPR or sensitivity is generally high, ranging from 98.50% to 100.0%. TPR represents the model’s ability to correctly identify positive instances (botnets) from the total number of actual positives. High TPR values indicate that the model is successful in identifying botnets. The AUC score, which represents the model’s overall ability to discriminate between positive and negative instances, is consistently high, with values ranging from 99.00 to 100.0%. A high AUC score suggests that the model is highly capable of distinguishing between botnet and non-botnet traffic.

**Table 9:** Models accuracy on different botnet datasets.

Dataset	Accuracy score (%)	Training accuracy (%)	Testing accuracy (%)	TPR (%)	AUC score (100%)
Bot-IoT^17^	100.00	100.00	100.00	100.00	100.00
CCC^45^	99.75	100.00	99.75	99.75	100.00
ISCX^19^	99.95	100.00	99.95	99.95	99.99
CTU-13^18^	98.88	98.99	97.84	98.77	99.01
CICIDS^47^	99.99	99.99	99.99	99.99	100.00
UNSW^94^	100.00	100.00	100.00	100.00	100.00

In our research, the underlying algorithm of focus is the ExtraTreesClassifier leverages an ensemble of de-correlated decision trees, which introduces both variance reduction and an increase in computational complexity. The core of the ExtraTreesClassifier’s complexity lies in the construction of N decision trees, where each tree is built from a bootstrap sample of the training data. The complexity of constructing a single decision tree is $O\left( {M * K * log K} \right)$, where M represents the number of training samples and K is the number of samples used at each node to determine the best split. In the case of the ExtraTreesClassifier, since the splits are chosen randomly and not from all possible splits, the complexity is reduced to $O\left( {M * log K} \right)$. In our experiments, we account for the feature selection process preceding the application of the ExtraTreesClassifier. The selection techniques employed, such as mutual information and PCA, add their own computational costs, which are respectively $O\left( {M * N} \right) and O\left( {N^{2} } \right)$, where N refers to the number of features. However, as these steps reduce the dimensionality of the problem, they can actually lead to a reduction in the subsequent computational burden during the training of the classifier. Table 10 delineates the computational expenditure entailed in deploying an ExtraTreesClassifier across a spectrum of botnet datasets, illustrating the model’s efficiency and resource utilization. It provides a meticulous account of the temporal demand for both the training and testing phases, measured in seconds, alongside a quantification of the memory resources requisitioned during these stages, presented in megabytes (MB). This comprehensive portrayal aids in discerning the practical implications of model deployment in varied operational environments, thereby facilitating informed decisions regarding the balance between computational costs and the performance efficacy of the model.

**Table 10:** Computational overhead of the model for different datasets.

Dataset	Training time (s)	Testing time (s)	Memory used for training (MB)	Memory used for testing (MB)
N-BaIoT^16^	28.4175	1.8934	1.5078	0.10547
Bot-IoT^17^	5.7186	0.3651	0.1718	0.0000
CCC^45^	1.1690	0.0220	10.511	1.3281
ISCX^19^	6.0245	0.4715	0.0117	0.0000
CTU-13^18^	46.2890	9.1598	32.4832	3.7632
CICIDS^47^	18.1896	0.5542	0.3871	0.8731
UNSW^94^	2.0460	0.0768	0.0312	0.0000

The ExtraTreesClassifier presents a trade-off between computational complexity and prediction accuracy. Despite the apparent high theoretical complexity, practical implementations benefit significantly from parallel computations. Our experimental setup and evaluations are designed to reveal the nuanced relationship between algorithmic complexity and model performance, ultimately guiding the user in selecting an appropriately balanced model for their specific real-world tasks.

**Figure 7:**
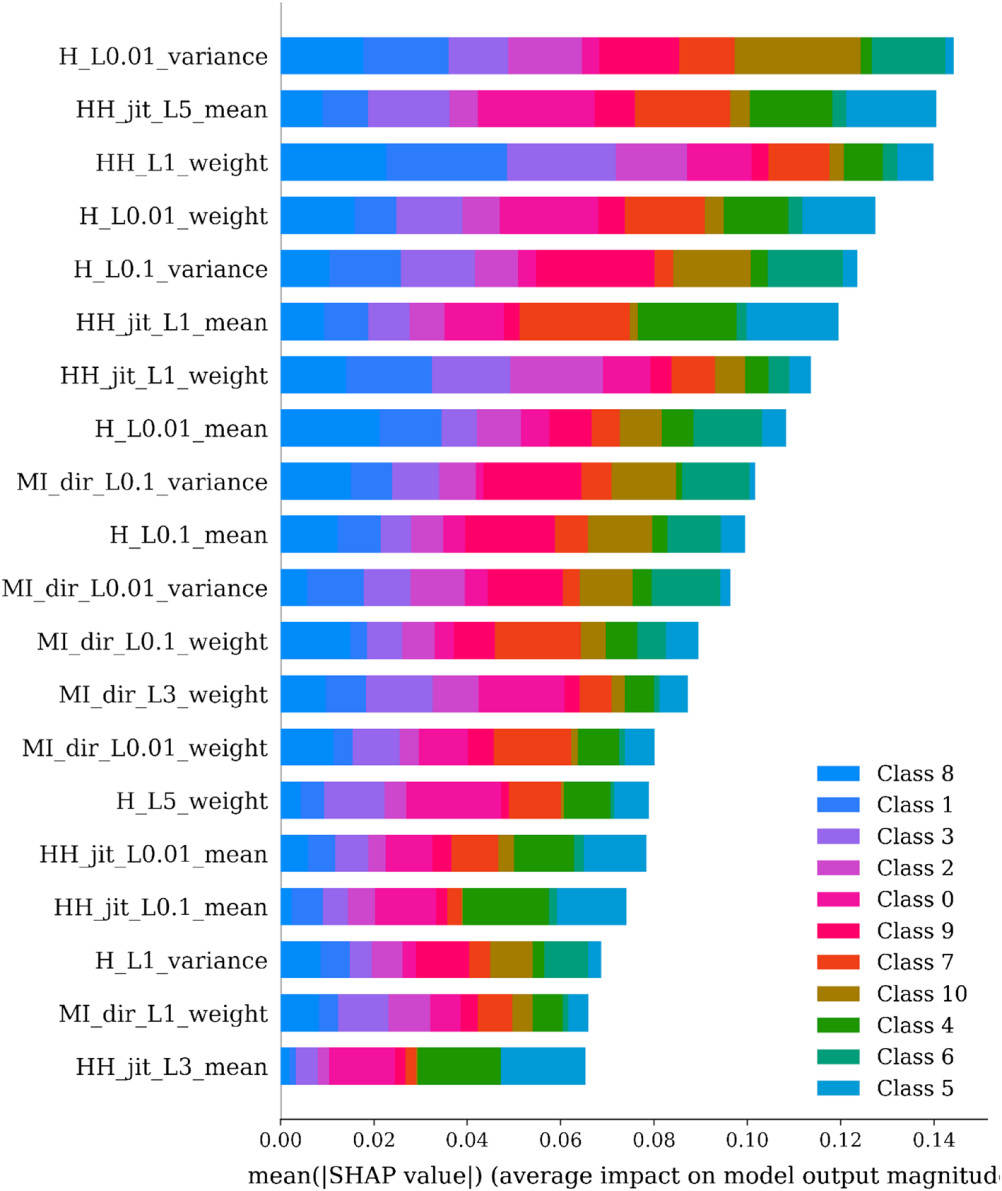
Mean SHAP values for feature importance across classes in ensemble model predictions.

**Figure 8:**
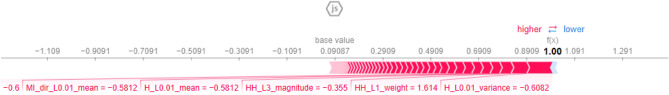
Detailed SHAP value impact on a single model prediction.

Figures 7 and 8 offer a compelling visualization of the interpretability aspect of our extra trees ensemble model’s predictions through the use of SHAP (SHapley Additive exPlanations) values. SHAP values provide a powerful framework for interpreting machine learning models by assigning an importance value to each feature for a particular prediction.

In Fig. 7, we observe a stacked bar chart detailing the mean SHAP values for different features across multiple classes. This visualization effectively communicates the average impact of each feature on the model output magnitude, allowing us to appreciate the relative importance of each feature in the classification process. The varied color coding corresponds to distinct classes, emphasizing the differentiated influence each feature exerts across various predicted classes. This nuanced depiction of feature impact is crucial in understanding how the extra trees ensemble model processes inputs to arrive at its decisions, enhancing trust in its predictions.

Figure 8 further enriches our interpretability discourse by presenting a SHAP value summary plot for a single prediction. This plot illustrates how each feature value contributes to the deviation from the base value (the model’s output value if no features had an effect) for a specific instance. Features pushing the prediction higher are shown in red, while those contributing to a lower prediction are in blue, providing a clear, intuitive understanding of the directionality and magnitude of each feature’s effect.

The positive aspect of these figures lies in their ability to convey the complexities of our ensemble model’s decision-making in an accessible and informative manner. Through these visualizations, we not only affirm the robust predictive power of our model but also its transparency, allowing for a deeper insight into the ‘why’ and ‘how’ behind its predictions. This interpretability is instrumental in validating the model's decisions and establishing a foundation for trust with end-users, making it an indispensable feature for real-world applications where understanding model predictions is as critical as their accuracy.

The proposed approach to feature selection is robust and multi-faceted, integrating correlation analysis, mutual information, and principal component analysis (PCA). This trifecta of techniques ensures a comprehensive understanding of feature relevance, capturing a broad spectrum of data characteristics that extend beyond the specifics of the training dataset. Such an inclusive feature selection methodology can be instrumental in identifying latent attributes indicative of botnet activity, thereby positioning the model to better generalize to new botnet variants. Moreover, our model harnesses the strength of an ensemble-based methodology, utilizing the ExtraTreesClassifier. Ensemble techniques, by their very nature, amalgamate insights from multiple decision trees, reducing the risk of overfitting to training data and increasing the chances of detecting previously unseen patterns. The ExtraTreesClassifier, in particular, employs a randomized and high-variance approach to feature selection and splits within individual trees, which provides a breadth of perspectives on the data. This diversity in model architecture makes it more adept at identifying outlying behaviors that could signify emerging threats. It is also noteworthy to highlight that ensemble methods have been shown to be effective in handling non-stationary environments, due to their capacity to build a consensus across varied learners. The performance of our model on multiple botnet datasets, as demonstrated in our research, speaks to its precision and generalizability, two attributes that are crucial for detecting new forms of botnet activities.

Overall, the proposed botnet detection model, incorporating hybrid feature selection and the extra trees ensemble classifier, consistently achieved high accuracy scores across various publicly available datasets. This success demonstrates the effectiveness of the suggested methodology in accurately identifying botnets in diverse scenarios, and it holds the potential to detect new and previously unseen types of botnets. Consequently, the model’s capabilities offer a substantial enhancement to computer system and network security by strengthening defenses against evolving botnet attacks.

## Conclusion

In the vanguard of cybersecurity, the escalation of botnet sophistication presents a formidable challenge, one that demands an equally evolved countermeasure. Our investigation, rooted in a pioneering blend of hybrid feature selection techniques and the prowess of the Extra Trees ensemble classifier, heralds a new epoch in botnet detection strategies. The model we have meticulously engineered not only achieves an unprecedented accuracy exceeding 99.99% but also maintains an exceptional True Positive Rate (TPR) of 99% alongside a virtually nonexistent False Positive Rate (FPR) of 0.00%. The result provides the model's unparalleled discernment in distinguishing between benign and malicious network behaviors. Further bolstered by the robustness across diverse datasets, the model stands as a paragon of versatility. Its consistent performance in various scenarios underscores its utility as a formidable tool in the real-world arsenal against cyber threats. The harmonious confluence of precision, recall, and F1-scores as evinced in our evaluation metrics bespeaks a balanced approach that mitigates the risks of overfitting while ensuring the retention of predictive power.

Network administrators and cybersecurity defenders can leverage this sophisticated detection model as a vigilant sentinel, guarding the sanctity of digital infrastructures. The adoption of this scheme promises a substantial elevation in network security and a stride forward in obviating the vulnerabilities that plague our interconnected systems.

Despite the pronounced efficacy of our model, we acknowledge certain limitations that pave the way for future enhancements. The integration of real-time analysis and the exploration of deep learning architectures could offer substantial improvements, bridging the gap between static detection and dynamic, adaptive defense mechanisms. Consequently, our future research trajectory is poised to not only iterate upon this framework but to revolutionize it, ensuring that it remains at the forefront of cybersecurity innovation.

## Data Availability

The research community has access to and can use the datasets used in this research. All pertinent details regarding these datasets are provided in the dedicated datasets section of this paper.

## Appendix: Features description of the N-BaIoT dataset

**Table:**

Feature title	Description
HpHp_L3_std	Standard deviation of the higher-layer protocol payload sizes in both directions
HpHp_L0.01_mean	Mean of the auto-correlation of higher-layer protocol payload sizes at time lag 0.01 s
H_L0.1_mean	Mean of the auto-correlation of packet sizes at time lag 0.1 s
H_L5_mean	Mean of the auto-correlation of packet sizes at time lag 5 s
HpHp_L0.1_radius	Radius of the auto-correlation of higher-layer protocol payload sizes at time lag 0.1 s
HH_L0.1_pcc	Pearson correlation coefficient between auto-correlation of packet sizes at time lag 0.1 s
HH_L5_mean	Mean of the auto-correlation of packet sizes at time lag 5 s
HpHp_L5_covariance	Covariance of higher-layer protocol payload sizes in both directions at time lag 5 s
H_L1_variance	Variance of packet sizes at time lag 1 s
HpHp_L3_mean	Mean of the auto-correlation of higher-layer protocol payload sizes at time lag 3 s
HH_L5_magnitude	Magnitude of the auto-correlation of packet sizes at time lag 5 s
HH_L0.1_weight	Weight of the auto-correlation of packet sizes at time lag 0.1 s
HH_L3_mean	Mean of the auto-correlation of packet sizes at time lag 3 s
HH_jit_L0.01_mean	Mean of jitter on packet sizes at time lag 0.01 s
HpHp_L0.01_covariance	Covariance of higher-layer protocol payload sizes in both directions at time lag 0.01 s
HpHp_L0.01_pcc	Pearson correlation coefficient between higher-layer protocol payload sizes at time lag 0.01 s
HH_L0.01_mean	Mean of the auto-correlation of packet sizes at time lag 0.01 s
MI_dir_L0.1_mean	Mean of the direct mutual information between features at time lag 0.1 s
HH_L5_std	Standard deviation of the auto-correlation of packet sizes at time lag 5 s
HH_L1_radius	Radius of the auto-correlation of packet sizes at time lag 1 s
HH_L3_pcc	Pearson correlation coefficient between auto-correlation of packet sizes at time lag 3 s
HH_L0.1_radius	Radius of the auto-correlation of packet sizes at time lag 0.1 s
HH_jit_L5_variance	Variance of jitter on packet sizes at time lag 5 s
HpHp_L0.01_std	Standard deviation of higher-layer protocol payload sizes in both directions at time lag 0.01 s
HH_jit_L1_mean	Mean of jitter on packet sizes at time lag 1 s
MI_dir_L1_mean	Mean of the direct mutual information between features at time lag 1 s
H_L3_variance	Variance of packet sizes at time lag 3 s
HH_L5_radius	Radius of the auto-correlation of packet sizes at time lag 5 s
H_L1_mean	Mean of packet sizes at time lag 1 s
HH_L0.1_mean	Mean of the auto-correlation of packet sizes at time lag 0.1 s
HH_L3_covariance	Covariance of the auto-correlation of packet sizes at time lag 3 s
HpHp_L0.1_weight	Weight of higher-layer protocol payload sizes in both directions at time lag 0.1 s
HH_L0.01_std	Standard deviation of the auto-correlation of packet sizes at time lag 0.01 s
HH_L3_std	Standard deviation of the auto-correlation of packet sizes at time lag 3 s
HH_L1_magnitude	Magnitude of the auto-correlation of packet sizes at time lag 1 s
HpHp_L5_pcc	Pearson correlation coefficient between higher-layer protocol payload sizes at time lag 5 s
HpHp_L1_covariance	Covariance of higher-layer protocol payload sizes in both directions at time lag 1 s
HH_jit_L3_mean	Mean of jitter on packet sizes at time lag 3 s
HH_jit_L0.1_mean	Mean of jitter on packet sizes at time lag 0.1 s
HpHp_L3_pcc	Pearson correlation coefficient between higher-layer protocol payload sizes at time lag 3 s
HH_L3_weight	Weight of the auto-correlation of packet sizes at time lag 3 s
MI_dir_L1_variance	Variance of the direct mutual information between features at time lag 1 s
HH_jit_L0.01_variance	Variance of jitter on packet sizes at time lag 0.01 s
HpHp_L1_magnitude	Magnitude of higher-layer protocol payload sizes in both directions at time lag 1 s
HH_L1_mean	Mean of the auto-correlation of packet sizes at time lag 1 s
HH_L3_magnitude	Magnitude of the auto-correlation of packet sizes at time lag 3 s
HpHp_L0.01_magnitude	Magnitude of higher-layer protocol payload sizes in both directions at time lag 0.01 s
HH_L0.01_radius	Radius of the auto-correlation of packet sizes at time lag 0.01 s
HH_L1_weight	Weight of the auto-correlation of packet sizes at time lag 1 s
HH_L1_std	Standard deviation of the auto-correlation of packet sizes at time lag 1 s
HpHp_L5_mean	Mean of higher-layer protocol payload sizes in both directions at time lag 5 s
HpHp_L0.01_weight	Weight of higher-layer protocol payload sizes in both directions at time lag 0.01 s
H_L0.01_mean	Mean of packet sizes at time lag 0.01 s
H_L0.1_weight	Weight of packet sizes at time lag 0.1 s
MI_dir_L3_mean	Mean of the direct mutual information between features at time lag 3 s
MI_dir_L0.01_weight	Weight of the direct mutual information between features at time lag 0.01 s
HH_L5_weight	Weight of the auto-correlation of packet sizes at time lag 5 s
H_L0.01_variance	Variance of packet sizes at time lag 0.01 s
HpHp_L0.1_pcc	Pearson correlation coefficient between higher-layer protocol payload sizes at time lag 0.1 s
HH_jit_L3_weight	Weight of jitter on packet sizes at time lag 3 s
HH_jit_L5_weight	Weight of jitter on packet sizes at time lag 5 s
MI_dir_L0.01_variance	Variance of the direct mutual information between features at time lag 0.01 s
MI_dir_L5_weight	Weight of the direct mutual information between features at time lag 5 s
HpHp_L0.1_magnitude	Magnitude of higher-layer protocol payload sizes in both directions at time lag 0.1 s
HH_L3_radius	Radius of the auto-correlation of packet sizes at time lag 3 s
HpHp_L5_radius	Radius of higher-layer protocol payload sizes in both directions at time lag 5 s
HH_L0.01_weight	Weight of the auto-correlation of packet sizes at time lag 0.01 s
H_L3_weight	Weight of packet sizes at time lag 3 s
MI_dir_L5_variance	Variance of the direct mutual information between features at time lag 5 s
HpHp_L3_weight	Weight of higher-layer protocol payload sizes in both directions at time lag 3 s
HH_L0.1_std	Standard deviation of the auto-correlation of packet sizes at time lag 0.1 s
H_L3_mean	Mean of packet sizes at time lag 3 s
H_L1_weight	Weight of packet sizes at time lag 1 s
HH_jit_L0.01_weight	Weight of jitter on packet sizes at time lag 0.01 s
HH_L5_covariance	Covariance of the auto-correlation of packet sizes at time lag 5 s
MI_dir_L3_weight	Weight of the direct mutual information between features at time lag 3 s
HpHp_L1_std	Standard deviation of higher-layer protocol payload sizes in both directions at time lag 1 s
HpHp_L0.1_std	Standard deviation of higher-layer protocol payload sizes in both directions at time lag 0.1 s
HH_L0.01_covariance	Covariance of the auto-correlation of packet sizes at time lag 0.01 s
HpHp_L0.1_covariance	Covariance of higher-layer protocol payload sizes in both directions at time lag 0.1 s
H_L5_variance	Variance of packet sizes at time lag 5 s
H_L5_weight	Weight of packet sizes at time lag 5 s
HpHp_L3_magnitude	Magnitude of higher-layer protocol payload sizes in both directions at time lag 3 s
HpHp_L3_radius	Radius of higher-layer protocol payload sizes in both directions at time lag 3 s
MI_dir_L5_mean	Mean of the direct mutual information between features at time lag 5 s
HH_jit_L3_variance	Variance of jitter on packet sizes at time lag 3 s
HH_L0.01_pcc	Pearson correlation coefficient between auto-correlation of packet sizes at time lag 0.01 s
HpHp_L1_weight	Weight of higher-layer protocol payload sizes in both directions at time lag 1 s
HH_jit_L5_mean	Mean of jitter on packet sizes at time lag 5 s
HH_L5_pcc	Pearson correlation coefficient between auto-correlation of packet sizes at time lag 5 s
HpHp_L0.1_mean	Mean of higher-layer protocol payload sizes in both directions at time lag 0.1 s
HpHp_L5_std	Standard deviation of higher-layer protocol payload sizes in both directions at time lag 5 s
HH_jit_L0.1_weight	Weight of jitter on packet sizes at time lag 0.1 s
HpHp_L1_pcc	Pearson correlation coefficient between higher-layer protocol payload sizes at time lag 1 s
HH_L0.1_magnitude	Magnitude of the auto-correlation of packet sizes at time lag 0.1 s
HpHp_L1_radius	Radius of higher-layer protocol payload sizes in both directions at time lag 1 s
HpHp_L1_mean	Mean of higher-layer protocol payload sizes in both directions at time lag 1 s
HH_L0.1_covariance	Covariance of the auto-correlation of packet sizes at time lag 0.1 s
HpHp_L5_weight	Weight of higher-layer protocol payload sizes in both directions at time lag 5 s
HH_L0.01_magnitude	Magnitude of the auto-correlation of packet sizes at time lag 0.01 s
HH_jit_L1_weight	Weight of jitter on packet sizes at time lag 1 s
MI_dir_L0.01_mean	Mean of the direct mutual information between features at time lag 0.01 s
HH_jit_L0.1_variance	Variance of jitter on packet sizes at time lag 0.1 s
H_L0.01_weight	Weight of packet sizes at time lag 0.01 s
MI_dir_L1_weight	Weight of the direct mutual information between features at time lag 1 s
HpHp_L0.01_radius	Radius of higher-layer protocol payload sizes in both directions at time lag 0.01 s
HH_L1_pcc	Pearson correlation coefficient between auto-correlation of packet sizes at time lag 1 s
H_L0.1_variance	Variance of packet sizes at time lag 0.1 s
HpHp_L3_covariance	Covariance of higher-layer protocol payload sizes in both directions at time lag 3 s
MI_dir_L0.1_variance	Variation in the direct mutual information between features with a 0.1 s time delay
MI_dir_L3_variance	Variance of the direct mutual information between features at time lag 3 s
HH_jit_L1_variance	Variance of jitter on packet sizes at time lag 1 s
MI_dir_L0.1_weight	Weight of the direct mutual information between features at time lag 0.1 s
HH_L1_covariance	Covariance of the auto-correlation of packet sizes at time lag 1 s
HpHp_L5_magnitude	Magnitude of higher-layer protocol payload sizes in both directions at time lag 5 s
